# Supplementary figures and images for: Inter-Cellular Forces Orchestrate Contact Inhibition of Locomotion
Source: Cell. 2015 Apr 9;161(2):361–73. doi: 10.1016/j.cell.2015.02.015 (PMC4398973; doi:10.1016/j.cell.2015.02.015)

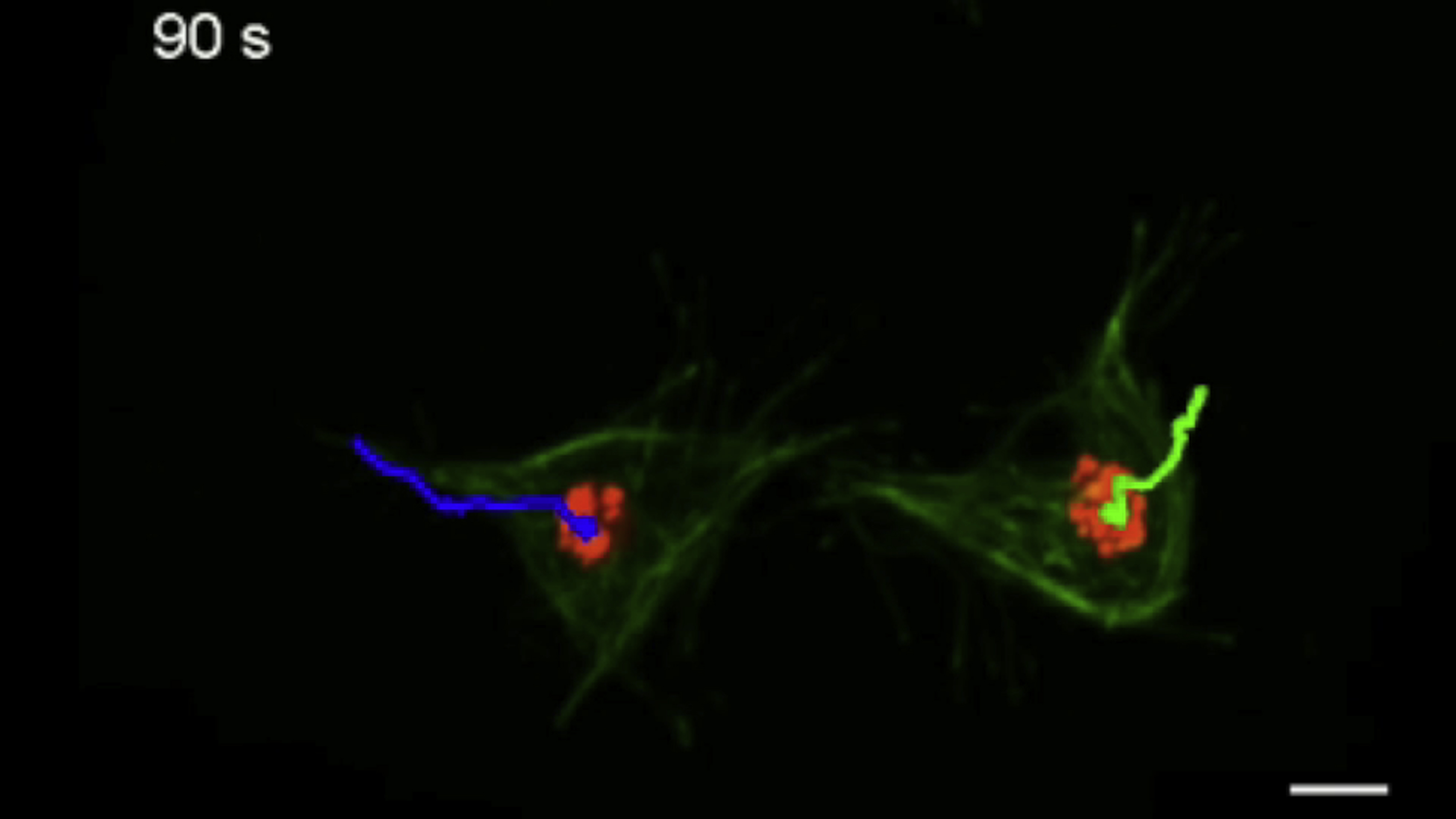

Supplement: Movie S1. Characterization of Hemocyte Collisions during Their Embryonic Dispersal, Related to Figure 1 — (00:00) Time-lapse movie of hemocyte developmental dispersal. Time-lapse movie of the developmental dispersal of Drosophila hemocytes beneath the ventral surface of an embryo. Hemocyte nuclei were labeled with a red marker. Hemocytes originally migrate down the ventral midline and subsequently disperse laterally until they acquire a uniform dispersal pattern. (00:25) Tracking hemocyte collisions. Hemocytes labeled with a nuclear marker (red) and a microtubule probe (green), were automatically tracked over an 8 min period to analyze the changes in motion during a collision. Time stamp is in reference to the point when microtubules first come into contact, which was used as a temporal register for subsequent kinematic analysis. Scale bar represents 5μm. (00:45) Average velocity of colliding hemocytes. The mean velocities of colliding partners during the collision time course. Note the synchronous changes in motion. (01:03) Visualization of actin and microtubules during CIL. Hemocytes labeled with an F-actin probe (magenta) and a microtubule marker (green) undergoing a collision. Time stamp is in reference to the point when microtubules first come into contact. Scale bar represents 5 μm. (01:19) Failure to undergo CIL during collision with a static cell or the rear of a migratory cell. A migrating hemocyte colliding with either a static cell (left panel) or the rear of another migratory cell (right panel) showing no cytoskeletal changes or repulsion. Hemocytes contain labeled F-actin. Note the formation of an actin cable (yellow arrowheads) in the right panel when the cell undergoes a lamella collision. Scale bar represents 5 μm. [file mmc1.jpg]

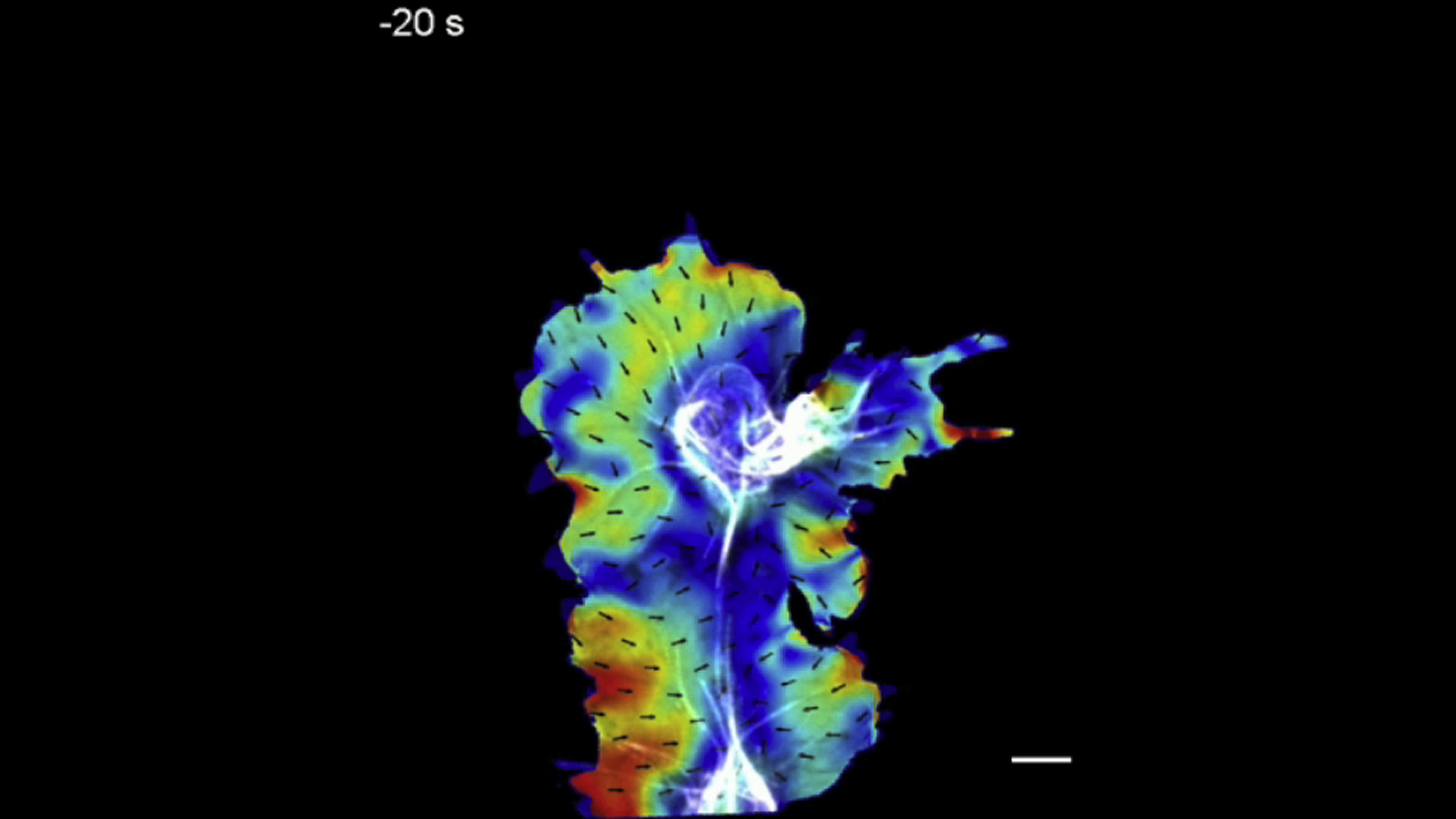

Supplement: Movie S2. Quantification of Actin Retrograde Flow in Freely Moving and Colliding Hemocytes, Related to Figure 2 — (00:00) Actin flow in freely moving hemocytes. Time-lapse movie of a freely moving wild-type hemocyte and the subsequent pseudo-speckle analysis of actin retrograde flow dynamics. Hemocytes have been labeled with an F-actin probe. The middle panels show the vector field of actin flow and the right panel the heatmap of flow velocity. Scale bar represents 5 μm. (00:12) Actin flow during CIL. Time-lapse movie of colliding hemocytes containing labeled F-actin (left panel) and the subsequent pseudo-speckle analysis of actin retrograde flow (right panel). Time stamp is in reference to the point when the lamellae come into contact. Scale bar represents 5 μm. (00:33) Simultaneous analysis of actin flow and microtubule dynamics during CIL. Pseudo-speckle analysis of actin retrograde flow in Movie S4 colocalized with microtubules (pseudo-colored white). Time stamp is in reference to the point when microtubules first come into contact. Scale bar represents 5 μm. (00:48) Heatmap of instantaneous changes in actin flow speed during CIL. Heat map of instantaneous changes in retrograde flow speed overlaid onto colliding hemocytes containing labeled F-actin. Note the sudden and synchronous increase in speed (red) during cell separation. Time stamp is in reference to the point of cell separation. Scale bar represents 5 μm. (00:59) Changes in actin flow direction during CIL. Left: pseudo-speckle analysis heatmap of actin retrograde flow in the lamella of a colliding hemocyte. Right: rose plot of actin flow direction in respect to the horizontal axis. Time stamp refers to the point when the lamellae first come into contact. [file mmc2.jpg]

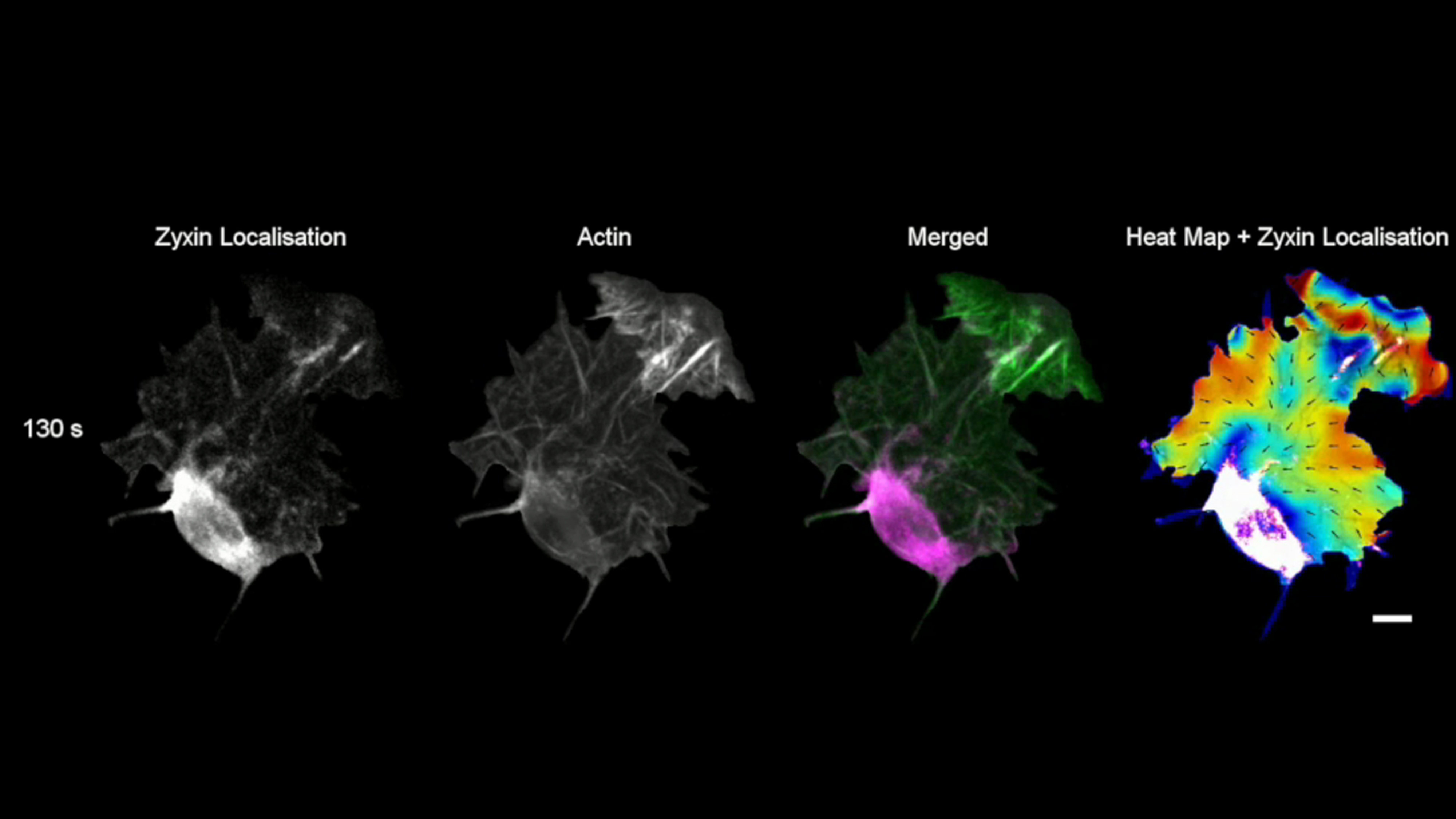

Supplement: Movie S3. Correlation of Actin and Microtubule Dynamics with Adhesion Formation during Collisions, Related to Figure 3 — (00:00) Colocalization Of Zyxin And Actin During CIL. Colocalization of Zyxin and actin during a collision. Right panel colocalizes Zyxin (pseudo-colored white) with the heatmap of actin retrograde flow. Note the slowing of the retrograde flow in a region in line with the Zyxin puncta. Time stamp is in reference to the point when the lamellae first come into contact. Scale bar represents 5 μm. (00:16) Colocalization of zyxin and microtubules during CIL. Colocalization of Zyxin and microtubules during a collision. Right panel shows a high-magnification movie of the microtubules targeting the Zyxin puncta. Time stamp is in reference to the point when microtubules first come into contact. Scale bar represents 5 μm. [file mmc3.jpg]

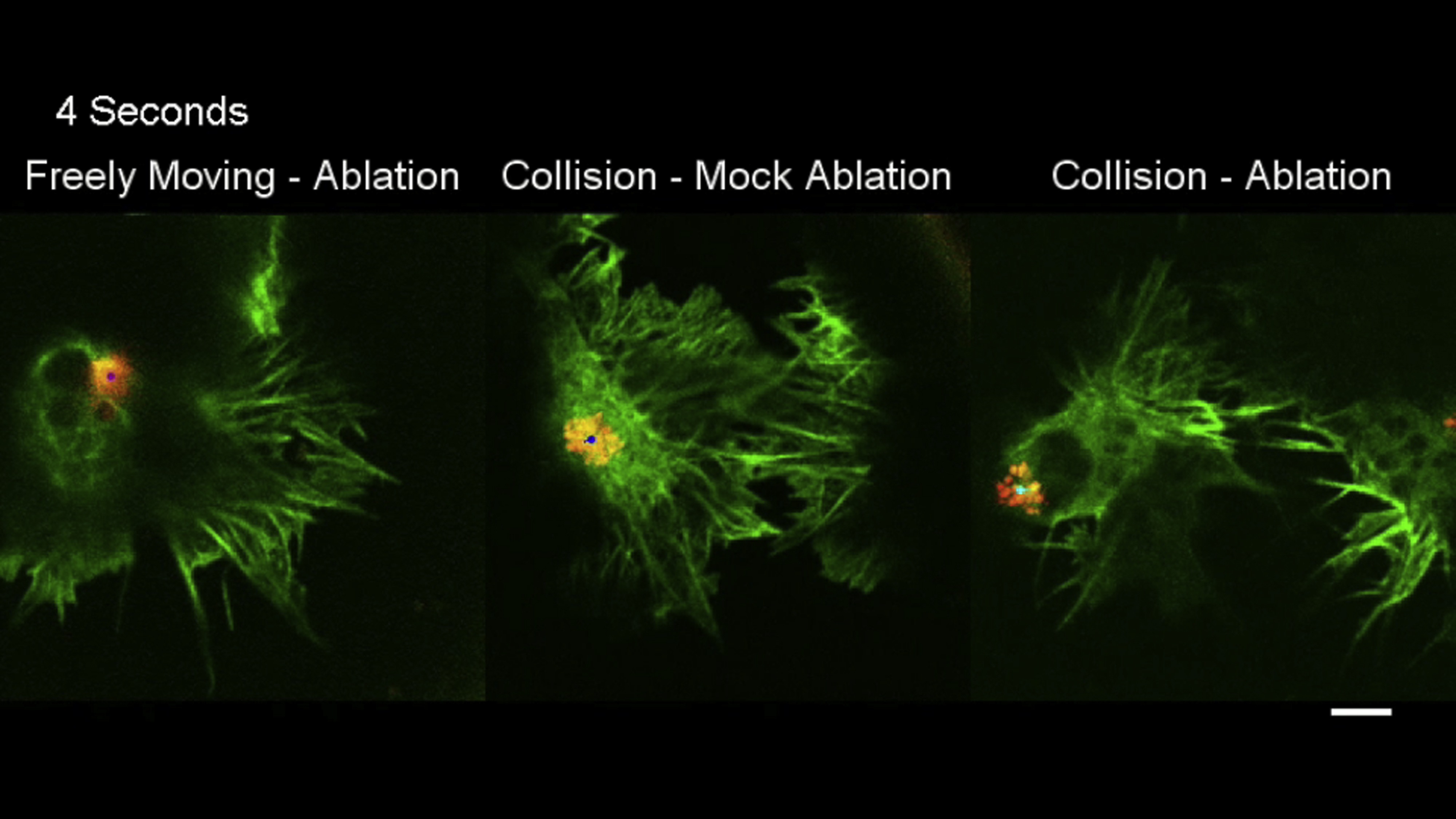

Supplement: Movie S4. Quantification of the Increase in Lamellar Tension during Hemocyte Collisions, Related to Figure 4 — (00:00) Lamellar recoil upon laser abscission during CIL. Analysis of lamellar recoil upon laser abscission. The recoil of the actin network (labeled with LifeAct-GFP) upon laser abscission was examined in freely moving and colliding cells (arrowhead highlights the ablation region). Ablation of the leading edge and the intracellular actin network of freely moving cells led to a small recoil of the network (left panels). In contrast, ablation of the overlap region of colliding cells across the actin fiber (right panel) led to a rapid and synchronous lamellar recoil. Scale bar represents 5 μm. (00:24) Analysis of cell movement upon laser abscission. The movement of the hemocyte cell body was examined after laser abscission (or mock ablation) in freely moving and colliding cells by tracking nuclear displacement (arrowhead highlights the ablation region). Ablation of the leading edge of a freely moving hemocyte led to no obvious movement of the cell, while after mock ablation the cell continued moving toward the colliding partner. In contrast, laser abscission of the region of lamellar overlap in colliding cells induced a sudden rearward movement of the cell body. Cells were labeled with LifeAct-GFP and an RFP nuclear marker. Scale bar represents 5 μm. (00:47) Modeling actin network stress during CIL. Modeling of actin-network stress in a colliding cell. Left: hemocyte containing labeled F-actin undergoing a collision. Right: heat map of modeled forces within the actin network. Note that before and after the collision, forces are distributed in a region adjacent to the cell body, while during cell contact, forces propagate to the region corresponding to the actin fiber. Red, high force; blue, low force. Time stamp is in reference to the point of cell separation. Scale bar represents 5 μm. [file mmc4.jpg]

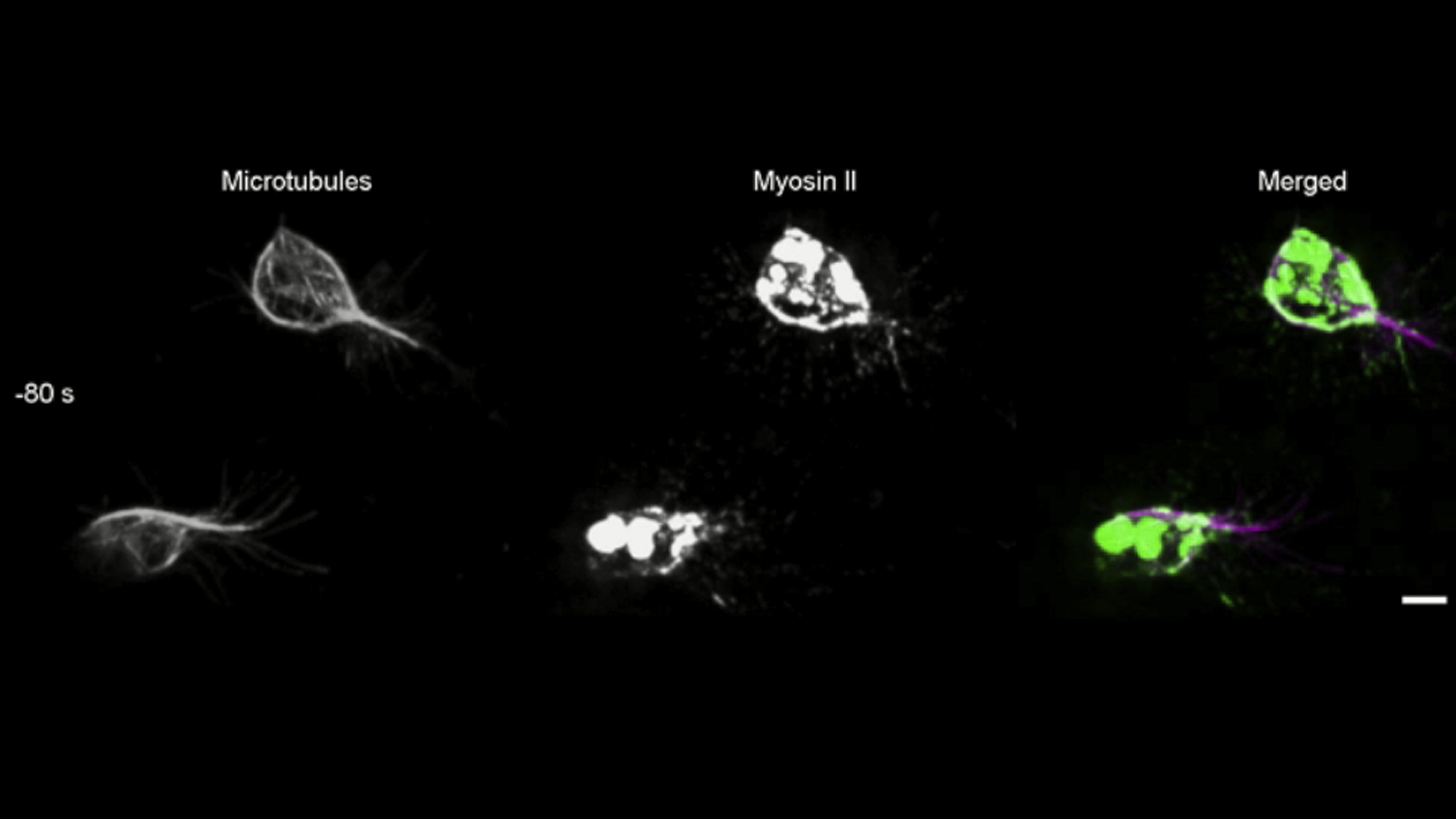

Supplement: Movie S5. Characterization of Myosin II Dynamics in Hemocytes, Related to Figure 5 — (00:00) Colocalization of Myosin II and actin during CIL. Colocalization of Myosin II and actin during a collision. Note that Myosin II moves in a retrograde fashion before and after collision, while during lamellae contact it colocalizes with the actin fiber. Time stamp is in reference to the point when the lamellae first come into contact. Scale bar represents 5 μm. (00:20) Colocalization of Myosin II and microtubules during CIL. Colocalization of Myosin II and microtubules during a collision. Time stamp is in reference to the point when microtubules first come into contact. Scale bar represents 5 μm. (00:33) Expression of constitutively actin diaphanous in a freely moving hemocyte. Constitutively active Diaphanous in a freely moving hemocyte. A constitutively active form of Diaphanous was expressed in a hemocyte containing labeled actin (magenta) and Myosin II (green). Note the enhanced localization of Myosin II in the lamella, which leads to a sudden contraction of the lamellar network and retraction fibers decorated with Myosin II. Scale bar represents 5 μm. [file mmc5.jpg]

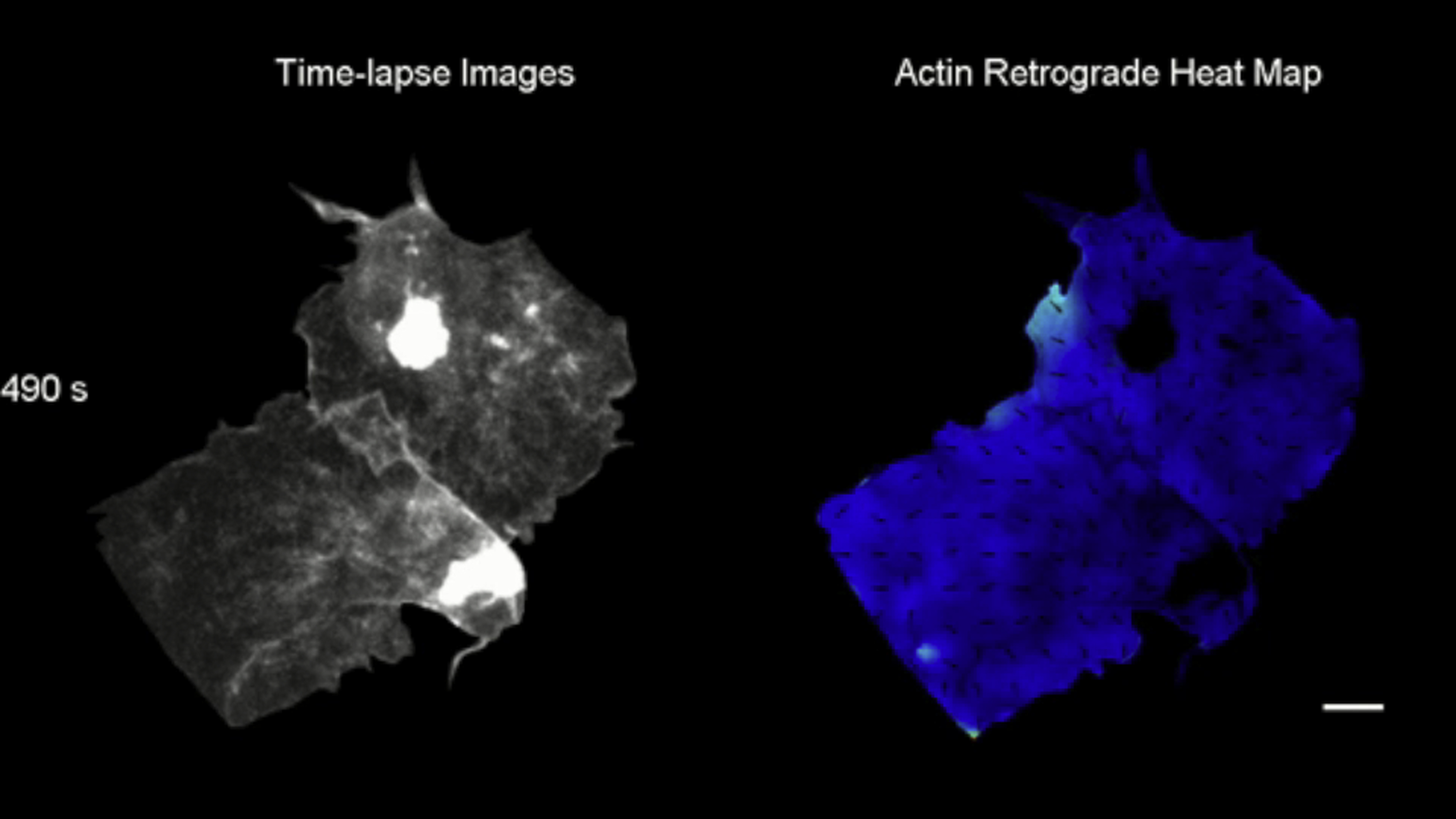

Supplement: Movie S6. Quantification of Actin Retrograde Flow in Freely Moving and Colliding Myosin II and Diaphanous Mutant Hemocytes, Related to Figure 6 — (00:00) Actin flow in freely moving Myosin II mutant cells. Left: freely moving myosin II mutant hemocyte containing labeled F-actin. Right: heat map of the actin retrograde flow. Note the significant reduction in actin flow compared Control cells in Movie S6. Scale bar represents 5 μm. (00:20) Actin flow in Myosin II mutant cells during CIL. Analysis of retrograde flow in myosin II mutant cells during a collision. Left: myosin II mutant hemocytes containing labeled F-actin undergoing a collision. Right: heatmap of the actin retrograde flow. Time stamp is in reference to the point when the lamellae first come into contact. Scale bar represents 5 μm. (00:46) Actin flow in freely moving diaphanous mutant cells. Left: freely moving diaphanous mutant hemocyte containing labeled F-actin. Right: heat map of the actin retrograde flow. Note that the distribution and speed of the flow is similar to control cells in Movie S6. Scale bar represents 5 μm. (01:02) Actin flow in diaphanous mutant cells during CIL. Analysis of retrograde flow in diaphanous mutant cells during collisions. Two examples of collisions that highlight the variability in the response. Left: diaphanous mutant hemocytes containing labeled F-actin undergoing a collision. Right: heat map of the actin retrograde flow. Note that in collision one, while there is some semblance of an actin fiber near the end of the response, there is no clear corridor of low retrograde flow. In contrast, in collision two there is a complete failure to form any actin fiber or corridor of low retrograde flow upon lamellae overlap. Time stamp is in reference to the point when the lamellae first come into contact. Scale bar represents 5 μm. [file mmc6.jpg]

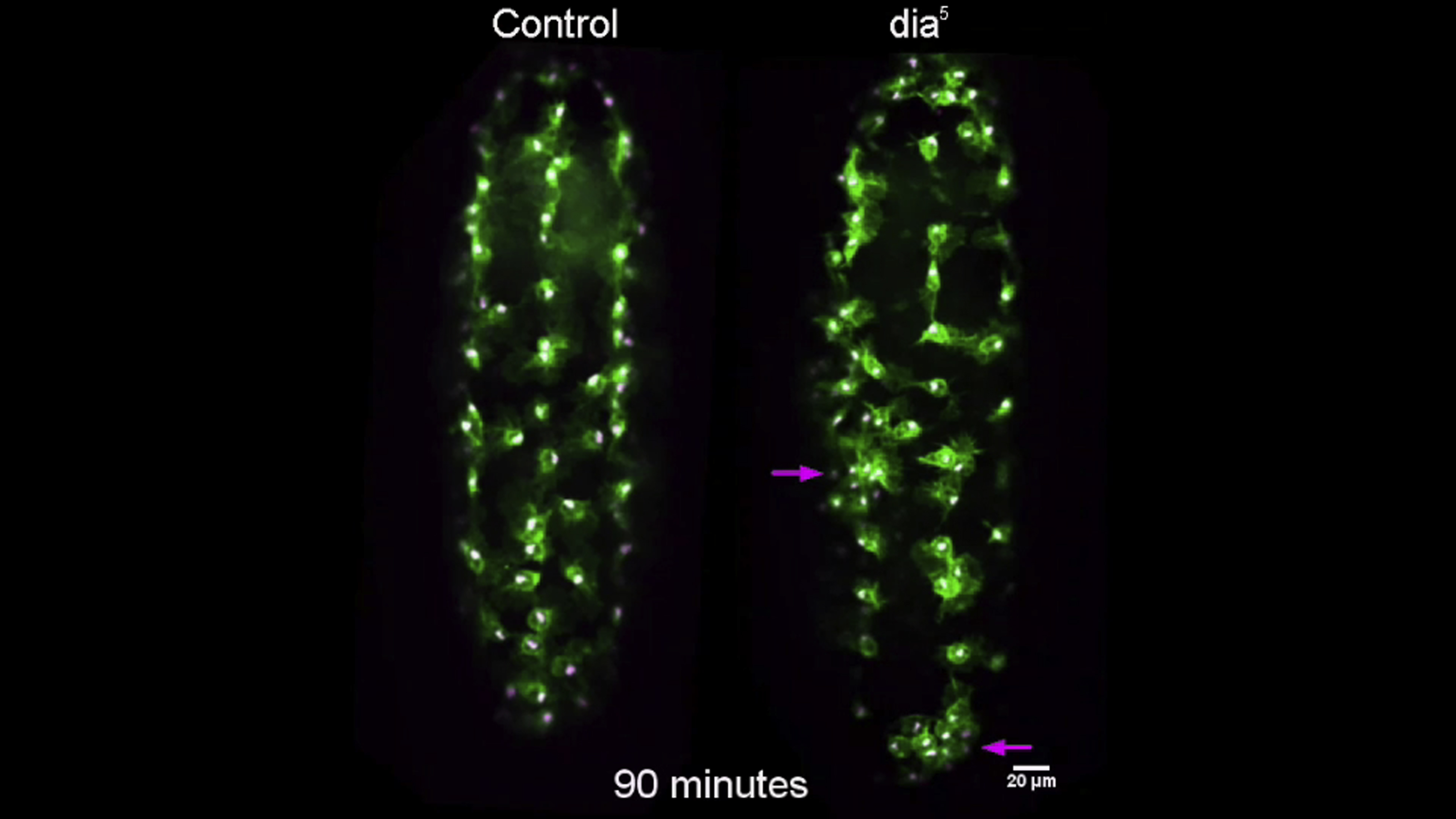

Supplement: Movie S7. Characterization of Real and Simulated Hemocyte Dispersal when Cells Undergo Random Repulsion, Related to Figure 7 — (00:00) Hemocyte dispersal in wild-type and diaphanous mutant embryos. Hemocytes containing labeled F-actin (green) and nuclei (magenta) migrating within the ventral surface. Note that while dia5 mutant hemocytes are capable of dispersal, they show regions of cell clumping (magenta arrows). Scale bar represents 20 μm. (00:23) Simulations of hemocyte dispersal comparing wild-type CIL parameters to randomized repulsion. Left: a simulation in which cells consistently take into account the direction of their colliding partners during repulsion. Right: a simulation in which cells are randomly responding to their colliding partners. [file mmc7.jpg]
