# Supplementary material for: Inter-Cellular Forces Orchestrate Contact Inhibition of Locomotion
Source: Cell. 2015 Apr 9;161(2):361–73. doi: 10.1016/j.cell.2015.02.015 (PMC4398973; doi:10.1016/j.cell.2015.02.015)
Supplement: Document S1. Article plus Supplemental Information [file mmc8.pdf]

Cell

Article

Inter-Cellular Forces Orchestrate Contact Inhibition of Locomotion

Graphical Abstract

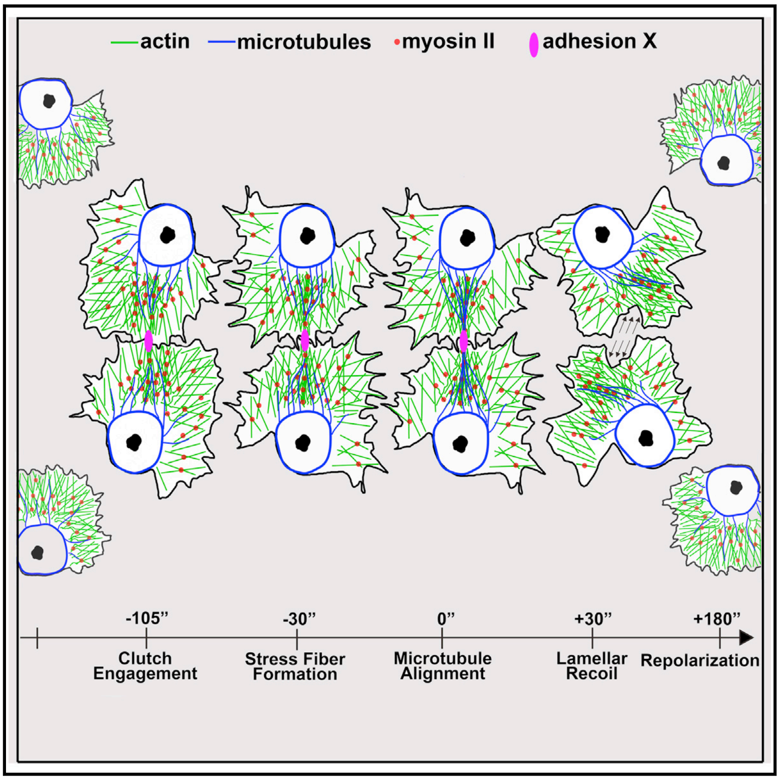

Authors

John R. Davis, Andrei Luchici, ..., Mark Miodownik, Brian M. Stramer

Correspondence

m.miodownik@ucl.ac.uk (M.M.), brian.m.stramer@kcl.ac.uk (B.M.S.)

In Brief

Contact inhibition of locomotion in *Drosophila* macrophages involves mechanical coupling of colliding actin networks. The resultant buildup in lamellar tension orchestrates the response and choreographs cell repulsion.

Highlights

- CIL in embryonic dispersing hemocytes requires choreographed changes in motility
- Tracking actin flow in vivo reveals synchronous changes in colliding actin networks
- An inter-cellular actin-clutch between colliding cells induces lamellar tension
- Tension buildup and release orchestrate the CIL response

Davis et al., 2015, Cell *161*, 361–373
April 9, 2015 ©2015 The Authors
http://dx.doi.org/10.1016/j.cell.2015.02.015

CellPress

Cell

Inter-Cellular Forces Orchestrate Contact Inhibition of Locomotion

John R. Davis,[1,5] Andrei Luchici,[1,2,5] Fuad Mosis,[1] James Thackery,[1] Jesus A. Salazar,[1] Yanlan Mao,[3] Graham A. Dunn,[1] Timo Betz,[4] Mark Miodownik,[2,*] and Brian M. Stramer[1,*]
[1]Randall Division of Cell and Molecular Biophysics, King's College London, London SE1 1UL, UK
[2]Department of Mechanical Engineering, University College London, London WC2R 2LS, UK
[3]Laboratory for Molecular Cell Biology, University College London, London WC1E 6BT, UK
[4]Centre de Recherche, Institut Curie, Paris, UMR168, France
[5]Co-first author
*Correspondence: m.miodownik@ucl.ac.uk (M.M.), brian.m.stramer@kcl.ac.uk (B.M.S.)
http://dx.doi.org/10.1016/j.cell.2015.02.015
This is an open access article under the CC BY license (http://creativecommons.org/licenses/by/4.0/).

SUMMARY

Contact inhibition of locomotion (CIL) is a multifaceted process that causes many cell types to repel each other upon collision. During development, this seemingly uncoordinated reaction is a critical driver of cellular dispersion within embryonic tissues. Here, we show that *Drosophila* hemocytes require a precisely orchestrated CIL response for their developmental dispersal. Hemocyte collision and subsequent repulsion involves a stereotyped sequence of kinematic stages that are modulated by global changes in cytoskeletal dynamics. Tracking actin retrograde flow within hemocytes in vivo reveals synchronous reorganization of colliding actin networks through engagement of an inter-cellular adhesion. This inter-cellular actin-clutch leads to a subsequent build-up in lamellar tension, triggering the development of a transient stress fiber, which orchestrates cellular repulsion. Our findings reveal that the physical coupling of the flowing actin networks during CIL acts as a mechanotransducer, allowing cells to haptically sense each other and coordinate their behaviors.

INTRODUCTION

Contact inhibition of locomotion (CIL), which is a cessation of forward movement upon migratory collision, is a process common to many cell types (Abercrombie and Heaysman, 1953; Astin et al., 2010; Dunn and Paddock, 1982; Gloushankova et al., 1998) that has recently been revealed to behave as a migratory cue for developmentally dispersing populations of cells during embryogenesis (Carmona-Fontaine et al., 2008; Davis et al., 2012; Stramer et al., 2010; Villar-Cerviño et al., 2013). This multifaceted phenomenon requires cells to specifically recognize each other, modulate their migratory capacity, and depending on the cell-type, subsequently repolarize. As a result of this complexity, the mechanisms behind CIL are largely unknown, and it is additionally unclear how these various behaviors during the process are integrated to induce a seamless response.

A range of inter-cellular adhesions and intracellular signaling pathways are postulated to be involved in CIL (e.g., Eph-ephrin [Astin et al., 2010], small GTPases [Carmona-Fontaine et al., 2008], planar cell polarity pathway [Carmona-Fontaine et al., 2008], and cell-cell adhesion [Gloushankova et al., 1998]). However, it is unclear exactly how these various signals feed into the cytoskeletal machinery to control the response. More crucially, nothing is known about the actin dynamics involved in CIL. As a central aspect of CIL is a rapid change in migration, it is clear that to understand the mechanisms behind this phenomenon it will be crucial to elucidate the dynamics of the actin network during the response.

During cell migration, the actin network provides the propulsion that allows a cell to generate movement. The actin cytoskeleton within the lamella of a migrating cell is in a constant state of retrograde flow. Actin polymerizes at the leading edge, which pushes the cell membrane forward. Subsequently, the force of polymerization against the membrane along with Myosin II driven contraction drives retrograde movement of the actin network; it is this treadmill that generates the forces behind cell motility. When a cell moves, cell-matrix receptors, such as integrins, become engaged and bind to the extracellular matrix. Integrin activation leads to a slowing of the actin flow at this integrin-based point of friction, and the force of the moving actin network is then transformed into extracellular traction stresses (Gardel et al., 2008). This integrin-dependent actin-clutch, and the resultant inverse correlation between actin flow and traction force, is hypothesized to be involved in the movement of numerous cell types.

We have been exploiting the embryonic migration of *Drosophila* macrophages (hemocytes) to understand the regulatory mechanisms of CIL and the function of this process during embryogenesis (Davis et al., 2012; Stramer et al., 2010). These cells develop from the head mesoderm and disperse throughout the *Drosophila* embryo taking defined migratory routes. One of these routes occurs just beneath the epithelium along the ventral surface where their superficial location in the embryo allows them to be imaged live at high spatio-temporal resolution approaching what can be achieved from cells in culture. This has revealed that hemocytes spread out to form an evenly

CrossMark

distributed pattern beneath the ventral surface within a thin acellular cavity (the hemocoel) (Stramer et al., 2010). We previously developed a mathematical model of hemocyte dispersal, and computer simulations revealed that this uniform cell spacing may be driven by contact inhibition (Davis et al., 2012). Indeed, a similar analysis of Cajal Retzius cell migration in the cerebral cortex showed an identical requirement for CIL in their dispersion (Villar-Cerviño et al., 2013), suggesting that CIL is a conserved mechanism capable of generating tiled cellular arrays.

Here, we show that hemocyte developmental dispersal requires precise contact inhibition dynamics. Quantification of changes in speed and direction during cellular collisions reveals that their CIL response is not stochastic but involves distinct kinematic stages that are synchronized between colliding partners. We also show that this choreographed movement involves a coordinated change in actin dynamics. Tracking actin flow within hemocytes in vivo reveals a physical coupling of the colliding actin networks through engagement of a transient inter-cellular adhesion. It is this ‘‘inter-cellular actin-clutch’’ and the coordinated build-up and release of lamellar tension in colliding cells that orchestrates their behaviors, allowing CIL to behave as an instructive migratory cue.

RESULTS

The Kinematic Steps of the CIL Response Are Synchronized in Colliding Hemocytes

Hemocytes disperse evenly within the ventral hemocoel during *Drosophila* embryogenesis and their CIL dynamics can be precisely analyzed during this process (Figure 1A; Movie S1) (Davis et al., 2012; Stramer et al., 2010). To elucidate the migratory phases of CIL, we first analyzed the changes in acceleration throughout the response with reference to the time of microtubule alignment between colliding hemocytes (Figure 1B; Movie S1), which we previously revealed is a hallmark of CIL that is associated with a change in hemocyte motility (Stramer et al., 2010). Our data revealed that there is a back acceleration upon microtubule alignment, signifying that cells were slowing down and/or changing direction (Figure 1C) (Davis et al., 2012). This was significant when calculated at either 60- or 20-s intervals (Figures 1C and S1A) highlighting that the time of microtubule alignment is correlated with a sudden change of motion during CIL.

The time of microtubule alignment allowed us to temporally register collisions and extend the time course of the acceleration analysis. We observed that 120 s before microtubule alignment there was a sudden forward acceleration, and 180 s after, an additional back acceleration event (Figure 1C). To determine whether these accelerations were due to changes in cell speed and/or direction, we quantified the internuclear distance of colliding cells during the CIL time course. Two minutes prior to microtubule alignment, the graph of internuclear distance over time revealed a sudden increase in slope (Figure 1D). This suggested that the cell speed increased, which was confirmed by analyzing the nuclear displacement rates (Figure 1E). Immediately upon microtubule alignment, the speed reduced (Figure 1E), which explained the sudden back acceleration (Figure S1A), and ~120 s later the nuclei moved apart (Figure 1D). Analysis of the SD of the internuclear distance over time also highlighted these stages by showing an abrupt decrease in variance as cells progressed from one phase to the next, suggesting that these stages were differentially regulated (Figure S1B). These distinct phases were also visualized by calculating the average velocity vector of left and right colliding cells (Movie S1), which additionally revealed the coordinated behavior of hemocytes during CIL.

As we previously revealed that the lamellae of colliding cells overlap before microtubule alignment (Stramer et al., 2010), we hypothesized that this initial interaction was instigating the kinematic changes. Indeed, lamellae of colliding cells made contact 105 ± 22 s prior to microtubule alignment (Figures 1F and 1G; Movie S1), coinciding with the forward acceleration phase of CIL. Furthermore, an actin fiber developed after lamellae contact that connected the colliding cells, which microtubules subsequently utilized as guides during alignment (Figure 1G). It is important to note that the formation of this actin fiber, and the subsequent repulsion, was not observed when a hemocyte contacted the rear of another cell (i.e., not the lamella), or collided with the lamella of a static cell (Movie S1). This analysis highlights that an interaction between lamellar actin networks of migrating cells is initiating the CIL response.

Analysis of the separation phase of CIL also revealed a synchronous response between colliding cells. Kymography of lamellar retraction revealed that colliding partners simultaneously retracted their lamellae at two to three times the speed of retraction events of freely moving cells (Figures 1H–1J). This retraction event initiated 32 ± 22 s after microtubule alignment, which coincided with the initiation of movement away from the colliding partner. The retraction of lamellae occurred prior to the development of new protrusions away from the colliding partner, suggesting that rapid lamellar retraction initiates cell repolarization (Figures S1C and S1D). Different cell-types exhibit distinct CIL behaviors, which are classified as either type 1 (involving contact-induced lamellar contraction) or type 2 (inhibition of locomotion without contraction) responses (Stramer et al., 2013). Our analysis suggests that hemocytes undergo a classical type 1 response similar in description to chick heart fibroblasts, which were also observed to exhibit sudden lamellar recoil (Abercrombie and Heaysman, 1953).

The Actin Cytoskeleton Rapidly Reorganizes in Colliding Partners during CIL

To understand how the actin networks were mediating the response, we analyzed the actin retrograde flow dynamics during CIL. Time-lapse movies of freely moving hemocytes, labeled with the actin probe, LifeAct-GFP, during their developmental dispersal revealed a highly dynamic actin network within their lamellae (Movie S2). We adapted a fluorescent pseudo-speckle tracking technique (Betz et al., 2009) to quantify the precise speed and direction changes of actin flow within hemocytes in vivo (Figure S2A; Movie S2). This revealed that freely moving hemocytes in vivo have a mean actin flow rate of 3.2 ± 1.8 μm/min, which is similar to growth cones in vitro (Betz et al., 2009).

Live imaging of collisions revealed significant reorganization of the actin networks during the response (Figure 2A; Movie S2). This reorganization coincided with the development of an actin fiber, which ran perpendicular to the leading edge, linking the

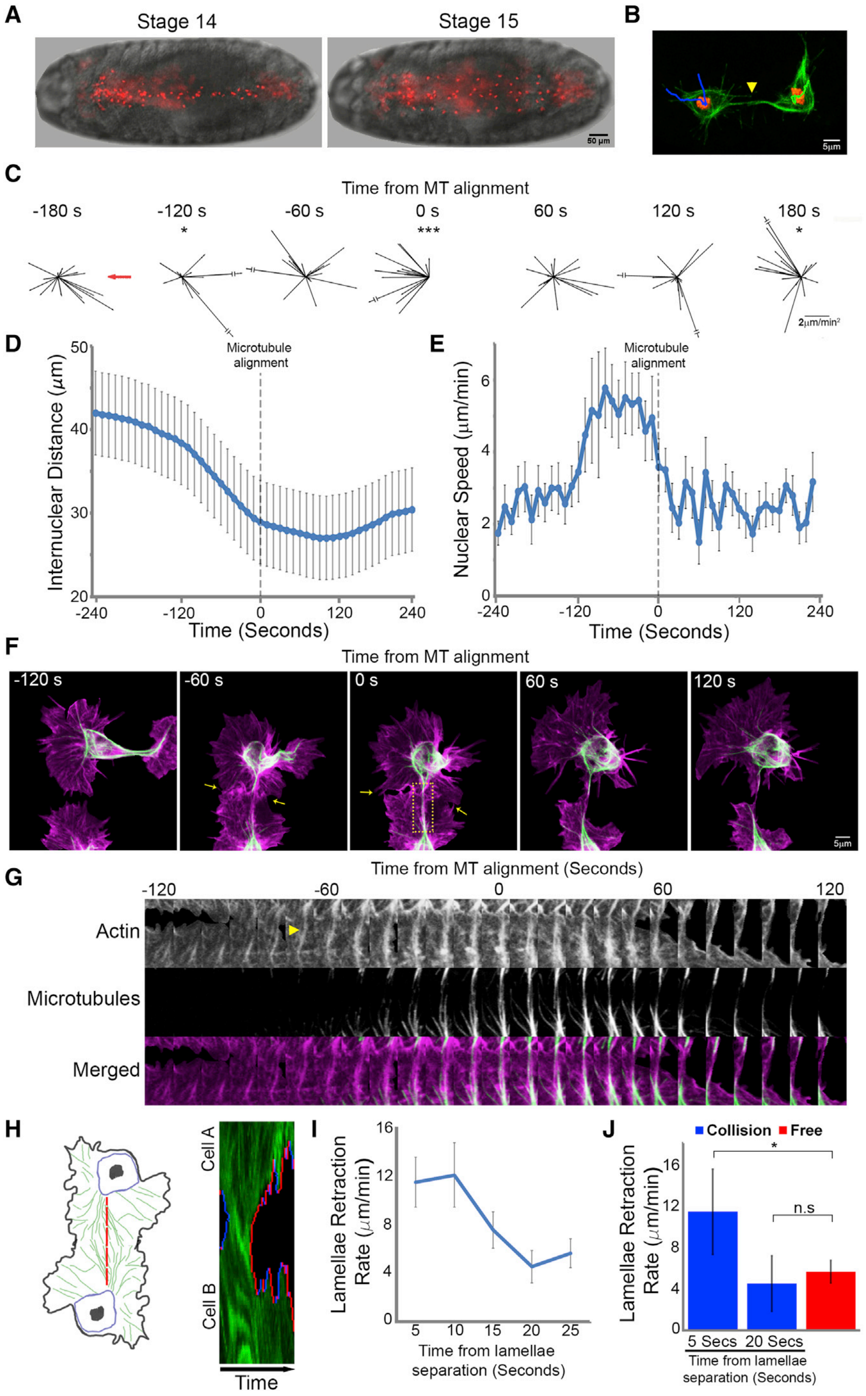

Figure 1. Hemocyte Contact Inhibition Involves Multiple Stages that Are Synchronous and Coordinated in Colliding Partners

(A) Dispersal of hemocytes labeled with a nuclear marker (red) beneath the ventral surface of a *Drosophila* embryo (bright-field) at developmental stages 14 and 15.

(B) Automatic tracking of nuclei (red) of colliding hemocytes while also registering collisions with microtubules (green). Time point of microtubule alignment (arrowhead) allows for temporal registration of CIL events in subsequent kinematic analyses.

(C) Time course of hemocyte accelerations (black arrows) surrounding a collision event with reference to the colliding partner (red arrow). All time points show random accelerations except at −120, 0, and 180 s where there is a bias along the x axis. $*p < 0.05$, $***p < 0.001$.

(D) Graph showing the internuclear distance of colliding cells during the CIL time course. Note the change in slope at −120, 0, and 120 s. Error bars represent SD.

(E) Graph showing nuclear speed during collisions. Note the increase in speed at −120 s and the subsequent decrease upon microtubule alignment. Error bars represent SD.

(F) Time-lapse sequence of colliding hemocytes labeled with an F-actin (magenta) and a microtubule (green) probe. Arrows highlight region of lamellae overlap.

(G) Kymograph of the region highlighted in (F) showing the time course of actin fiber formation (arrowhead highlights the initial development of the actin fiber) and microtubule alignment.

(H) Kymograph of lamellar activity (red regions show lamellar retraction and blue extension) in colliding partners along the actin fiber (red dotted line in schematic). Note that retraction is simultaneous in colliding cells upon lamellae release.

(I) Quantification of the rate of lamella retraction over time. Error bars represent SEM.

(J) Quantification of lamella retraction rates at 5 and 20 s after cell separation compared with average retraction rates in freely moving cells. Error bars represent SD. $*p < 0.05$.

See also Figure S1 and Movie S1.

lamellae of colliding cells (Figures 2A and S2B). Pseudo-speckle microscopy of collisions highlighted a slowing of the actin flow within a corridor that colocalized with the actin fiber (Figure 2A; Movie S2) and the aligned microtubule bundle (Figure 2B; Movie S2). Quantification of the actin flow rate within the region surrounding the actin fiber revealed a decrease in magnitude during the response, which suddenly increased upon lamellae separation (Figures 2C, 2D, and S2C; Movie S2). It is interesting to note that these analyses highlight that the increase in actin flow speed occurred in two phases; there was an abrupt spike immediately upon lamellae separation lasting ~20 s (that coincided with the duration of lamella recoil) (Figure 1I), followed by an additional increase during cellular repolarization (Figures 2D and S2C). Analysis of instantaneous changes in flow direction also revealed an increase in rotation after lamellae contact (Figure 2E), which rapidly returned to levels observed in freely moving cells after lamellae separation (Figure 2F; Movie S2). The change in flow direction coincided with a movement of actin fibers within the lamella toward the nascent actin cable, which contributed to its formation (Figure S2D). Upon lamellae

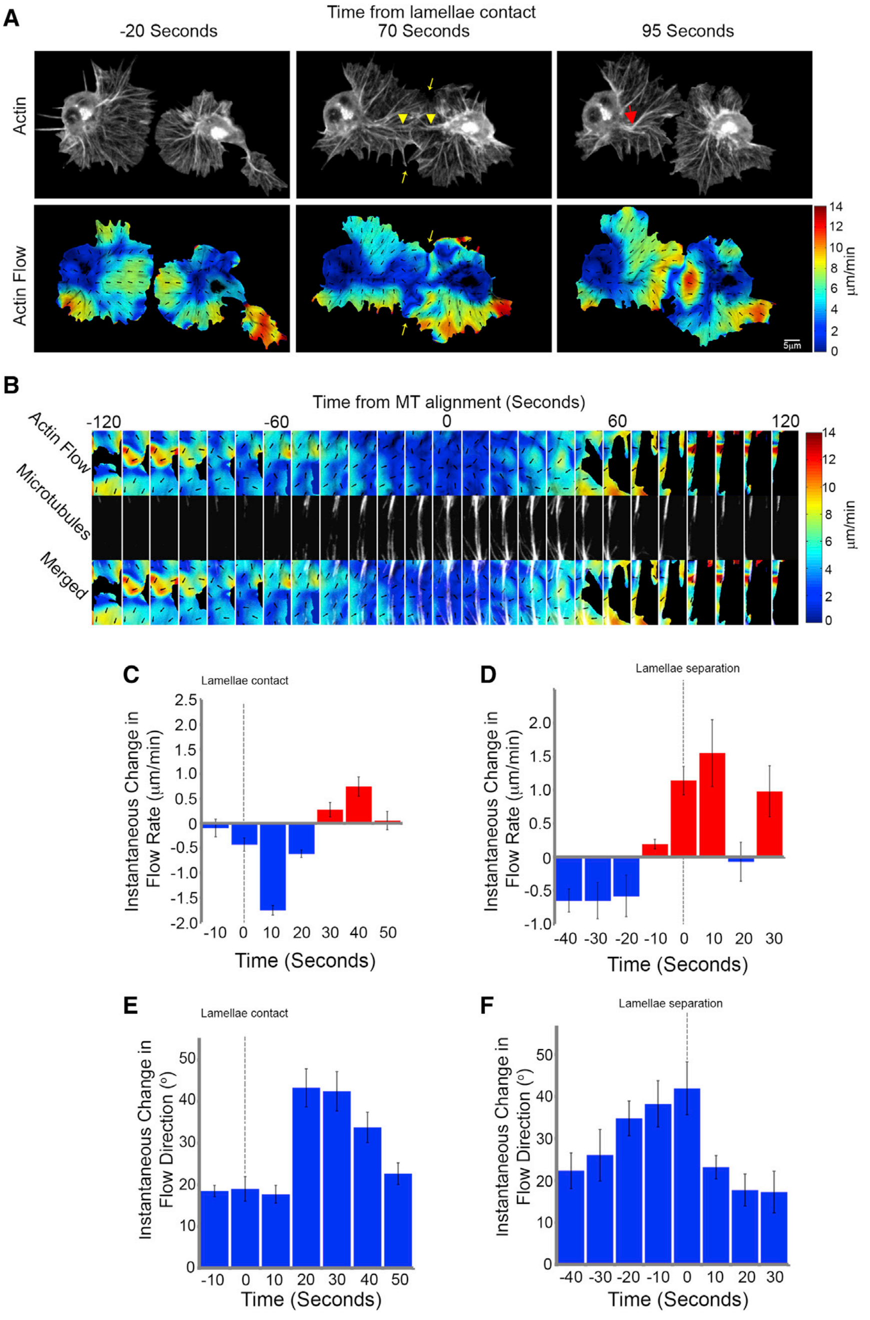

Figure 2. During Contact Inhibition, the Actin Network Is Rapidly Reorganized in Colliding Partners

(A) Top panels are still images from a time-lapse movie of hemocytes containing labeled F-actin during a collision. While cells are in contact, an actin fiber develops between the cell body and the point of contact in colliding partners (arrowheads), which often deforms and breaks upon lamellar retraction (red arrow). Bottom panels highlight actin flow dynamics obtained from the pseudo-speckle analysis. Note that the decreased actin flow in the vicinity of lamellae overlap (highlighted by yellow arrows) is due to the inability of the algorithm to distinguish between the two networks.

(B) Kymograph of the region surrounding the actin fiber highlighting the actin retrograde flow dynamics and the alignment of the microtubule bundles (pseudocolored white).

(C and D) Instantaneous changes in retrograde flow rate quantified from lamellae contact (C) or lamellae separation (D).

(E and F) Instantaneous changes in retrograde flow direction quantified from lamellae contact (E) or lamellae separation (F).

For (C)–(F), error bars represent SD. See also Figure S2 and Movie S2.

separation, the actin fiber deformed and was subsequently lost as the actin flow rapidly returned to its normal retrograde direction (Figure 2A; Movie S2). These data highlight that CIL involves a dramatic reorganization of the lamellar actin network.

Development of a Transient Cell-Cell Adhesion during CIL Coincides with a Coordinated Reorganization of the Actin Network

As a number of cell types have been reported to form transient inter-cellular adhesions during CIL (Gloushankova et al., 1998; Theveneau et al., 2010), we wanted to determine whether a cell-cell adhesion was responsible for the rapid and seemingly coordinated actin network changes in colliding hemocytes. As Zyxin is known to be a marker of both cell-matrix and cell-cell adhesions (Hirata et al., 2008), we expressed mCherry-Zyxin in hemocytes and colocalized Zyxin-labeled adhesions with actin during CIL. Immediately upon lamellae overlap, a punctum of Zyxin developed at the site of cell-cell contact and persisted for the duration of the response (Figures 3A and 3B; Movie S3). Subsequently, the actin fiber formed immediately behind this concentration of Zyxin (Figures 3A and 3B; Movie S3). We hypothesized that Zyxin foci represented transient cell-cell adhesions that modulate actin retrograde flow in a process analogous to the integrin-based actin-clutch reported in migrating cells in vitro (Gardel et al., 2008). Indeed, visualization of Zyxin while analyzing actin flow revealed that after development of the Zyxin puncta the retrograde flow rate decreased within a corridor immediately behind (Figures 3C and 3D; Movie S3). Furthermore, microtubules polymerized toward this site of adhesion (Figure 3E; Movie S3). Immediately upon microtubule targeting of this adhesion, Zyxin levels decreased (Figure 3F).

The development of an inter-cellular adhesion during CIL suggested that the colliding actin networks were becoming physically coupled. We therefore examined whether this coupling could lead to synchronous changes in actin retrograde

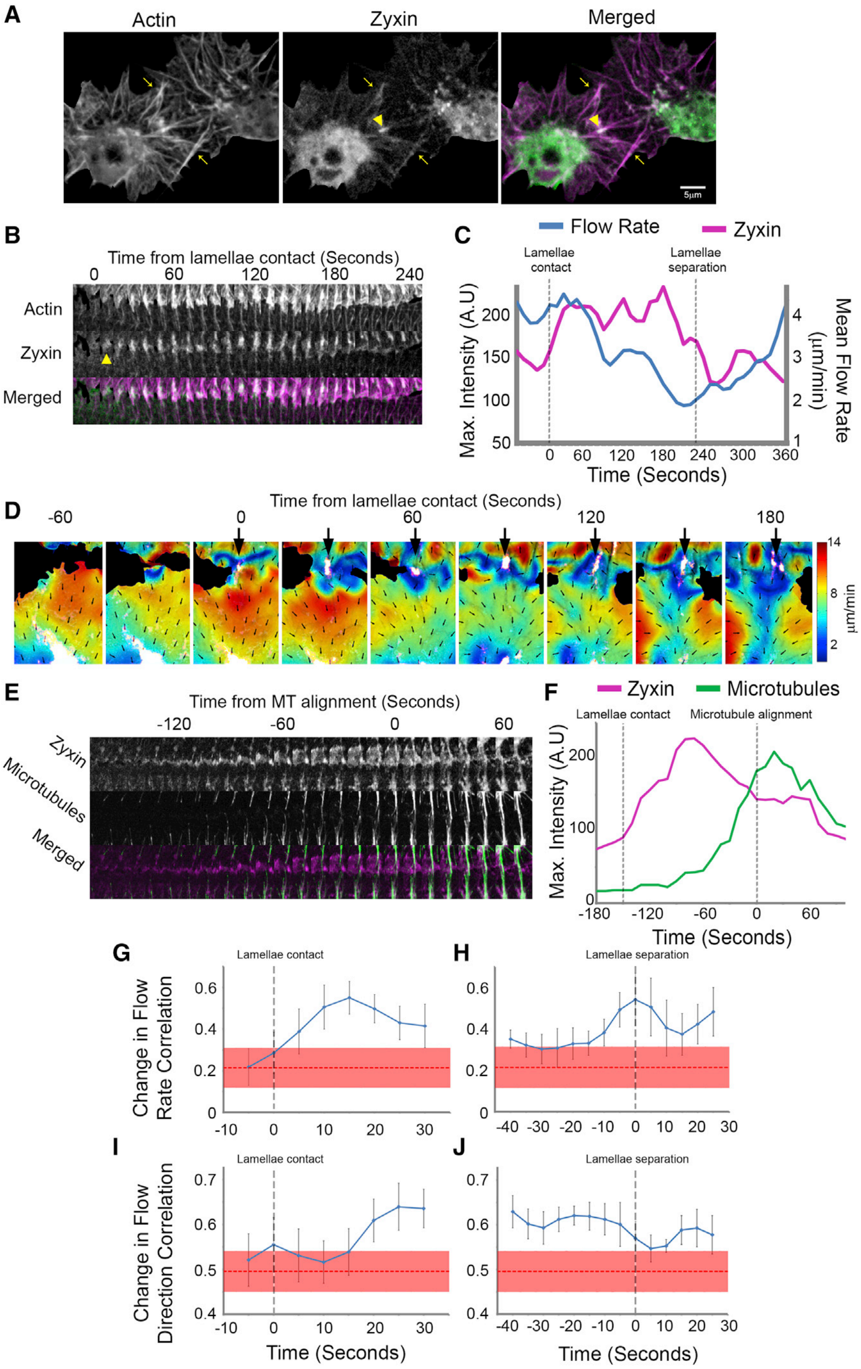

Figure 3. Actin Network Reorganization Correlates with the Formation of a Transient Cell-Cell Adhesion

(A) Still image of a collision between hemocytes expressing mCherry-Zyxin (green) and labeled F-actin (magenta), which highlights the inter-cellular adhesion at the point of initial contact (arrowhead). Arrows highlight region of lamellae overlap.

(B) Kymograph of Zyxin and actin dynamics in the region of the actin fiber. Note that the punctum of Zyxin forms in line with the actin fiber and persists for the duration of the time in contact (arrowhead highlights the initial formation of the punctum).

(C) Quantification of the maximum intensity of Zyxin and average actin flow rate during the collision.

(D) Analysis of actin flow dynamics in comparison with Zyxin localization (pseudocolored white). Note that the region of low retrograde flow develops in line with the inter-cellular adhesion (arrows).

(E) Kymograph of Zyxin and microtubule dynamics in the region of the actin fiber highlighting microtubule targeting of the Zyxin puncta.

(F) Maximum intensity of Zyxin and microtubules at the inter-cellular adhesion in the region highlighted in (E).

(G–J) Cross correlation of the instantaneous changes in flow rate (G and H) and flow direction (I and J) in lamellae of colliding cells. Error bars represent SEM. Red dotted lines represent the mean correlation between colliding cells immediately prior to cell-cell contact with the thickness representing the SEM.

See also Movie S3.

flow in colliding cells. Investigation of the correlation between instantaneous changes in flow speed in colliding partners revealed an increase immediately upon lamellae overlap, which slowly diminished as cells remained in contact (Figure 3G). Subsequently, at the time of lamellae separation there was an additional abrupt increase in the correlation of instantaneous changes in flow speed (Figure 3H; Movie S3). Similarly, correlation between the instantaneous changes in flow direction of colliding partners showed an increase ~20 s after lamellae contact, which remained high until the time of separation (Figures 3I and 3J). These data reveal that, similar to the orchestrated motion of colliding cells, the actin networks are behaving in a coordinated fashion during CIL.

Increase and Redistribution of Actin Network Stress during Cell Collision

The sudden and synchronous retraction event that occurred upon cell separation (Figure 1H) suggested that tension is developing within the actin network during CIL. We therefore analyzed lamellar tension by laser abscission of the actin cytoskeleton as the recoil rate of the network is indicative of tension within the lamella. Ablating the leading edge or an actin fiber within the lamellae of a freely moving cell led to an initial recoil rate of 28.6 ± 6.8 and 33.2 ± 4.3 μm/min, respectively. Interestingly, the recoil was unidirectional toward the cell body when the ablation was performed within the lamella (Figures 4A and 4B; Movie S4), which may be explained by a bias of myosin contraction toward the rear of the lamella (Svitkina et al., 1997; Yam et al., 2007). In contrast, cutting the region of lamella overlap across the actin fiber linking colliding

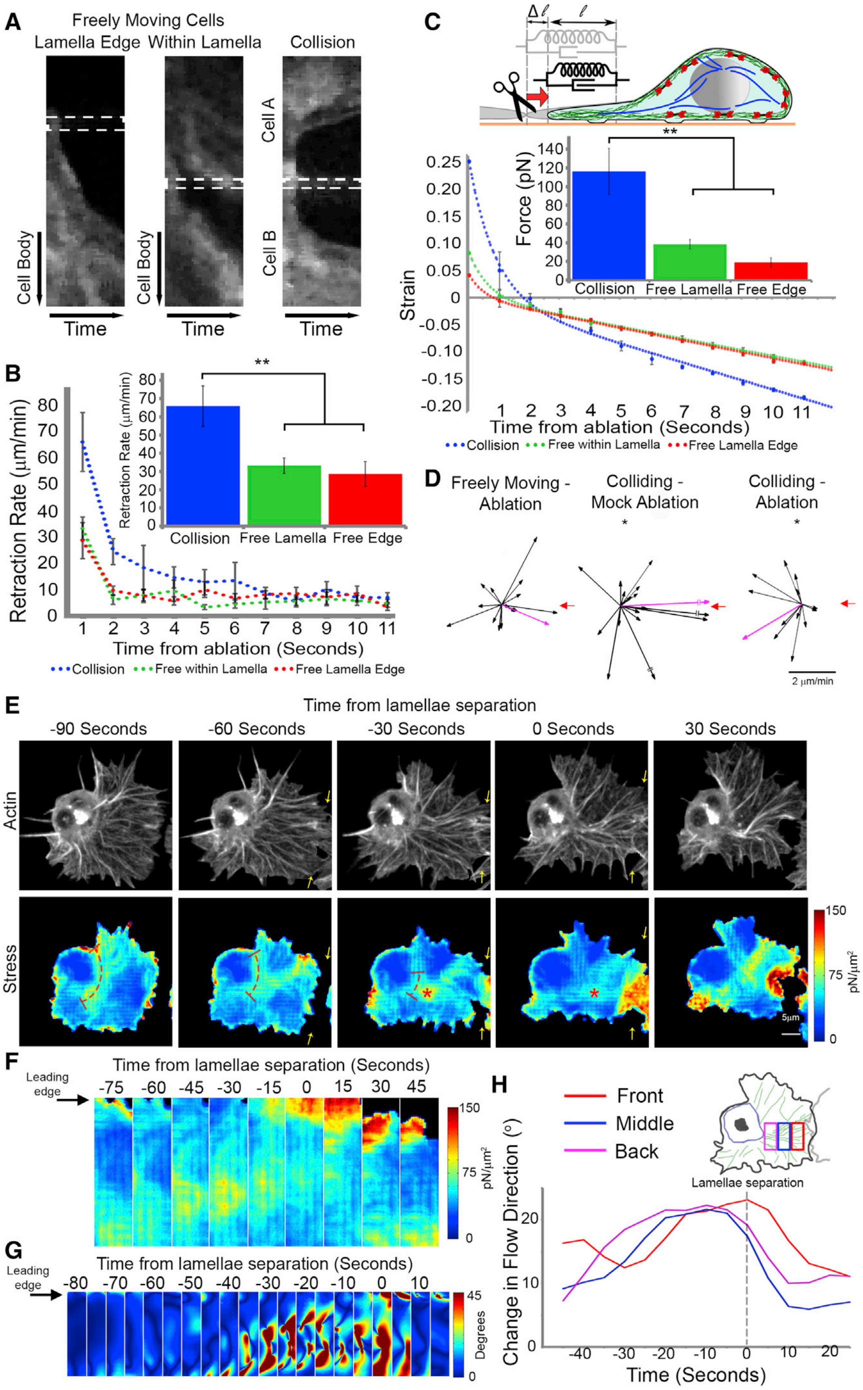

Figure 4. Lamellar Stresses Are Increased and Redistributed during CIL

(A) Kymographs of lamellar recoil upon laser abscission of the actin network in freely moving and colliding cells. Dotted rectangle highlights the width of the ablation region.

(B) Quantification of recoil rate over time and initial recoil rate upon laser abscission. Error bars represent SEM. **p < 0.01.

(C) Quantification of lamellae strain over time upon laser abscission and modeled forces assuming that the actin network behaves elastically over short time scales. The elastic and dissipative mechanical properties in the lamellae are modeled by an exponential decay of the strain that is overlaid onto the constant retrograde flow. Note that zero strain represents the end of the exponential decay. Assuming mechanical properties similar to previously published lamellae we can estimate the tension. Inset: Sketch illustrating the mechanical model of an elastic and dissipative element. The strain u is calculated by the ratio Δl /l. Error bars represent SEM. **p < 0.01.

(D) Hemocyte velocities in freely moving and colliding cells 60 s after laser abscission with respect to the ablation site (red arrow). Magenta arrow is the average direction of the population. Note that after mock ablation there was a significant forward movement of cells, while ablation of the fiber during cell collision led to a significant rearward movement. *p < 0.05.

(E) Localization of actin network stress during cell collision. Top panels: a time-lapse series of a hemocyte containing labeled F-actin undergoing a collision (adapted from Figure 2A). Bottom panels: modeled intracellular actin stresses. Note that stresses were only measured for regions of the lamella that persisted for a 40-s period as deformation history is required in the analysis. Arrows highlight region of lamellae overlap. Dotted line highlights the redistribution of stresses around the cell body and asterisks the regions of high stress that colocalize with the actin fiber.

(F) Kymograph of lamellar stresses over the region that colocalized with the actin fiber. Note the redistribution of stress from the back of the network to the front.

(G) Kymograph of the instantaneous changes in actin flow direction in the region colocalizing with the actin fiber.

(H) Quantification of the mean change in flow direction of the actin network in three regions corresponding to the back, middle, and front of the actin fiber. Note that the changes initially increase in the rear of the network.

See also Movie S4.

cells led to a significantly enhanced retraction rate of 65.8 ± 11.1 μm/min suggesting increased tension was stored within the actin network during CIL (Figures 4A and 4B; Movie S4). We subsequently modeled the amount of force present within the actin network by assuming that the actin cytoskeleton behaves elastically over short time scales (Gardel et al., 2004). Tracking edge displacement of the lamellae upon laser abscission allowed us to measure the changes in strain of the lamellar network as it retracts. This revealed that laser abscission of the lamellae leads to an initial rapid exponential decay that is caused by the sudden release of lamellar tension, followed by a slower linear phase as the retrograde actin flow continuously pulls in the network (Figure 4C). Assuming a lamellar stiffness similar to growth cones, the measured strain upon the release of the

lamellar tension allowed us to infer the forces present within the actin network in freely moving and colliding cells. This revealed that forces in colliding lamellae were ~3-fold higher than freely moving cells (Figure 4C). Furthermore, release of this tension by laser abscission during cell collision was sufficient to induce a rearward movement of the cell body away from the ablation site (Figure 4D; Movie S4).

We also adapted previously developed techniques to infer the localization of stresses that drive the retrograde flow using an estimation of actin network deformation (Betz et al., 2011; Ji et al., 2008). As previous correlation of flow changes suggested that the colliding actin networks were becoming coupled (Figures 3G–3J), the model assumed that the colliding networks were behaving as a single visco-elastic material. Interestingly, this analysis revealed that there was a redistribution of stresses during the response. Prior to cell-cell contact, most of the stress was localized around the cell body similar to freely moving hemocytes. However, upon collision the stress redistributed from around the cell body to the base of the actin fiber (Figure 4E; Movie S4) where it subsequently propagated to more distal regions of the fiber (Figure 4F). After cell separation, the stress became localized again to the region around the cell body as in freely moving cells (Movie S4). This stress redistribution indicated that actin cytoskeletal changes were spreading, not from the site of lamellae contact, but from the rear of the network. Indeed, when we examined the distribution of instantaneous changes in the direction of actin flow, we observed a similar propagation from the cell rear (Figures 4G and 4H). These data confirm that lamellar tension increases during CIL and propagates from the rear of the network, suggesting that these cytoskeletal changes are not initiated by a local signal released from the site of cell contact.

Coupling of Colliding Actin Networks Leads to the Development of a Transient Stress Fiber

As Myosin II is the major motor responsible for generating contraction within the actin cytoskeleton, we examined its dynamics during hemocyte migration. In freely moving cells, both actin and Myosin II flow in a similar retrograde fashion (Figures 5A, 5B, S3A, and S3B). However, comparable to our previously observed changes in actin flow direction during CIL (Movie S2), Myosin II flow reoriented perpendicularly toward the actin fiber (Figures 5C, 5D, and S3C; Movie S5). The actin fiber developed coincidentally with Myosin II accumulation along its length (Figures 5E and 5F) and subsequently became decorated with repeating puncta of Myosin II ~1.4 μm apart (Figure S3D), similar to stress fibers in vitro (Hotulainen and Lappalainen, 2006). Furthermore, the microtubule bundle aligned along the Myosin II decorated actin fiber (Movie S5). Analogous to the propagation of modeled stresses from the rear of the actin fiber, Myosin II intensity first increased at the back of the lamella (Figure 5G) and colocalized with the corridor of reduced retrograde flow (Figure 5H). Upon lamellae retraction, the Myosin II puncta along the actin fiber rapidly moved in a retrograde fashion with the actin network toward the cell body (Movie S5).

We also examined the localization of the formin, Diaphanous (Dia), which has previously been shown to decorate stress fibers within cells in vitro and be important for stress fiber assembly (Nakano et al., 1999; Sandbo et al., 2013). Expression of constitutively active Dia within hemocytes led to reduced cell spreading and an accumulation of Myosin within the lamella, with periods of severe contraction of the actin network suggesting that activation of Dia can increase lamellar tension (Figure S3E; Movie S5). As *Drosophila* Dia is also known to be involved in filopodia formation (Homem and Peifer, 2009), it was unsurprising to observe wild-type Dia localized to filopodia in freely moving cells (Figures S3F and S3G). However, upon cell collision, Dia localized along the actin fiber (Figures 5I and 5J). These data suggest that the previously described actin fiber is a stress fiber-like structure that couples colliding actin networks during CIL.

Myosin II-Dependent Contraction and Stress Fiber Formation Are Essential for Coordinating the CIL Response in Colliding Cells

We subsequently examined the importance of lamellar contraction and stress fiber formation in regulating the CIL response. We first analyzed actin flow in hemocytes of embryos mutant for *myosin II heavy chain* (hereafter mentioned as Myosin II and called Zipper [Zip] in the fly). *Drosophila* zygotic mutant embryos (*zip^1*) only begin to show defects at late stages of embryogenesis (when we also begin to image hemocyte motility) suggesting that maternal levels of the protein perdure to this stage of development (Young et al., 1993). Mutant hemocytes were initially capable of migrating from the head where they originate, but began to fail in motility soon after this stage (Figure S4A). This reveals that, in contrast to the reported dispensability of Myosin II in cell migration in 2D environments in vitro (Doyle et al., 2012), Myosin II is critical for hemocyte motility during embryogenesis. Analysis of retrograde flow in *myosin II* mutants showed that they had a significant reduction in speed (1.5 ± 1.0 μm/min) (Figures S4B and S4C; Movie S6). Expression of a GFP-tagged Myosin II specifically in hemocytes within *zip^1* mutants rescued their developmental dispersal (Figure S4A) and retrograde flow rates (Figures S4B and S4C), showing that Myosin II is critical for actin flow in vivo. These data suggest that *myosin II* mutant hemocytes have a reduction in the contractility of their actin networks.

We subsequently examined the lamellar dynamics of *myosin II* mutant hemocytes during collisions. Time-lapse movies of *zip^1* hemocyte collisions revealed that colliding cells failed to reorganize their actin networks (Figure 6A), or increase their lamellar retraction rates upon separation (Figures 6B and 6C; Movie S6). They also did not develop a prominent actin fiber during cell collision (Figure 6D). These defects were accompanied by a failure to cease their forward motion upon collision and an increased time in contact during the CIL response (Figures 6E and 6F). However, similar to fibroblasts in vitro (Giannone et al., 2007), loss of Myosin II in freely moving hemocytes also reduced their rate of lamellar retraction, showing that they had general defects in lamellar tension (Figure 6C).

We therefore specifically analyzed the role of the stress fiber during CIL by examining the response in *diaphanous* (*dia^5*) mutants. In contrast to *zip^1* hemocytes, freely moving *dia^5* mutant cells showed no aberration in actin retrograde flow (Figures S5A–S5C; Movie S6), cell migration (Figures S5D and S5E), or rate of lamellar retraction (Figure 6I). However, colliding cells

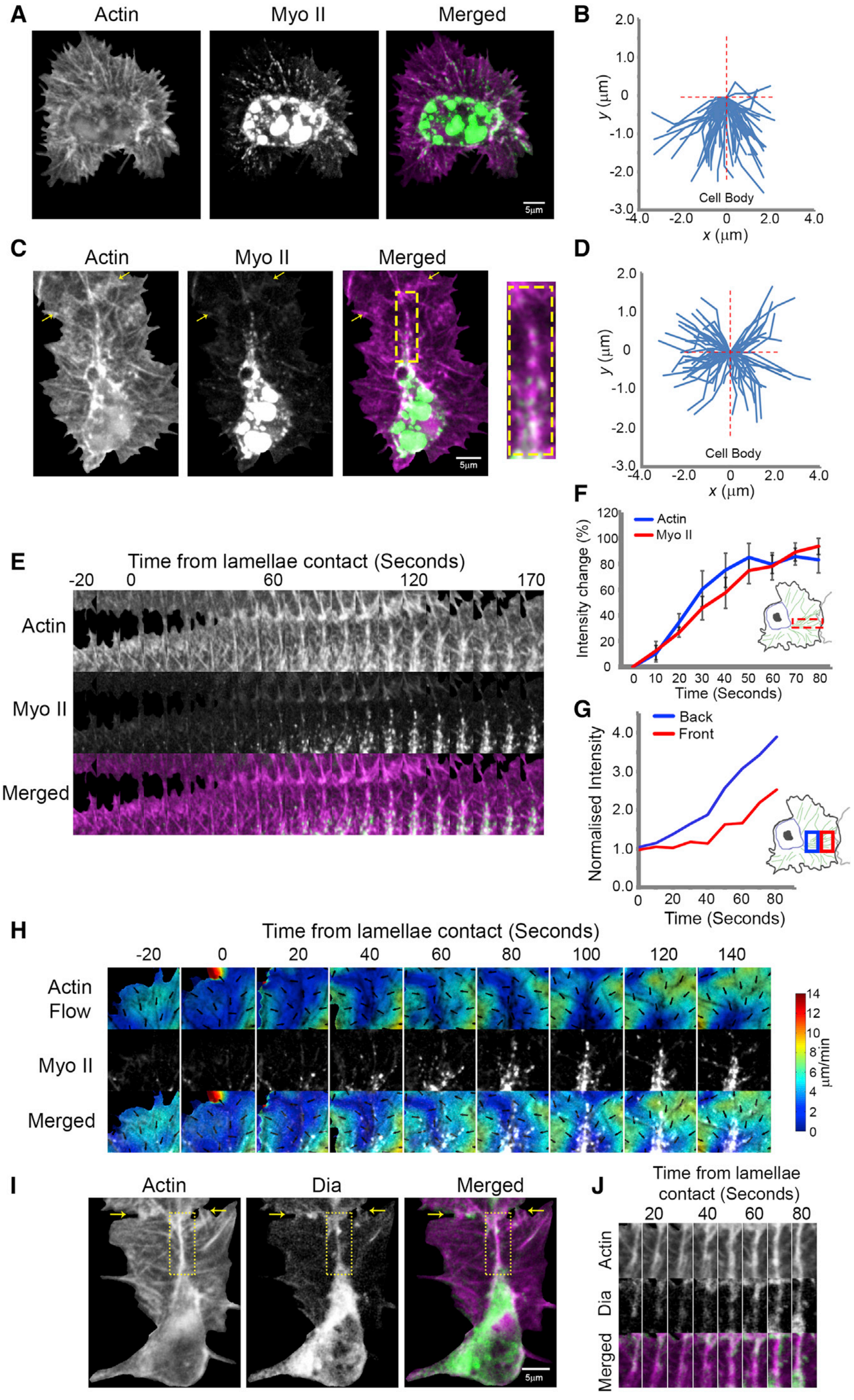

Figure 5. The Actin Fiber that Couples Colliding Cells Is a Stress Fiber-like Structure

(A) Still image of a freely moving hemocyte containing labeled F-actin (magenta) and Myosin II (green).

(B) Quantification of Myosin II tracks in freely moving cells.

(C) Still image of a collision between hemocytes containing labeled actin and Myosin II. Note the puncta of Myosin II along the actin fiber (inset). Arrows highlight region of lamellae overlap.

(D) Quantification of Myosin II tracks for 40 s upon lamellae overlap during CIL.

(E) Kymograph of the region surrounding the actin fiber in (C) highlighting Myosin II accumulation during a collision.

(F) Quantification of the increase in actin and Myosin II intensity in the region corresponding to the actin fiber relative to values prior to lamellae contact. Error bars represent SEM.

(G) Quantification of Myosin II intensity in regions corresponding to the back versus the front of the actin fiber during CIL.

(H) Analysis of actin flow dynamics in comparison with Myosin II localization (pseudocolored white). Note that actin network reorganization precedes Myosin II accumulation along the stress fiber.

(I) Still image of a collision between hemocytes containing labeled actin (magenta) and Diaphanous (green). Arrows highlight region of lamellae overlap.

(J) Kymograph of the region surrounding the actin fiber in (I).

See also Figure S3 and Movie S5.

showed a highly variable response (Movie S6) and, on average, failed to generate an actin fiber (Figure 6D) or coordinate their actin dynamics (Figures 6G and S5F–S5I). Additionally, their lamellar retraction rates upon collision were no different from freely moving cells (Figures 6H and 6I). Furthermore, similar to *zip*^{1} hemocytes, *dia*^{5} mutant cells showed a reduced capacity to cease their forward movement upon collision (Figure 6E) and persisted in contact for a longer duration (Figure 6F). Despite these defects, *dia*^{5} hemocytes, similar to wild-type cells, formed a Zyxin puncta and showed some microtubule alignment during collision (Figure S6). These data reveal that preventing stress fiber formation results in an aberrant response whereby cells eventually separate, but in the absence of the sudden lamellar retraction characteristic of type 1 CIL.

A Coordinated CIL Response Is Essential for Hemocyte Dispersal

As *dia*^{5} mutant hemocytes showed uncoordinated cytoskeletal dynamics during collisions (Figures S5G and S5I), we wanted to determine whether this defect also led to an uncoordinated kinematic response during CIL. Analysis of the acceleration time course revealed that, in contrast to the three acceleration changes observed in wild-type hemocytes (Figure 1C), *dia*^{5} mutant cells only showed the back acceleration upon microtubule alignment (Figure 7A). However, this back acceleration was significant only when calculated at 60-s

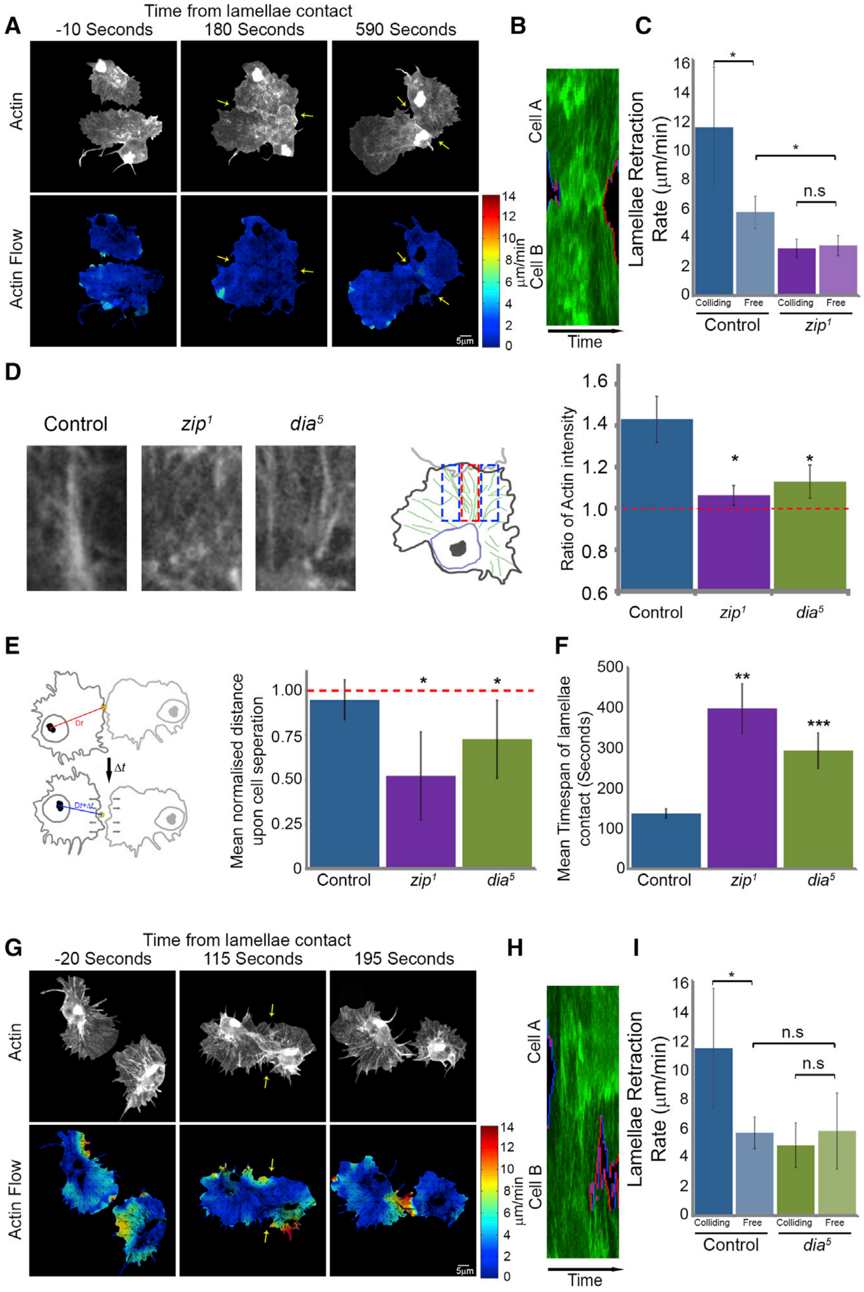

Figure 6. A Stress Fiber-like Structure Is Required for a Normal CIL Response

(A–C) Myosin II mutant (*zip*^{1}) collisions. (A) Top panels are still images from a time-lapse movie of hemocytes containing labeled F-actin during a collision. Bottom panels are heatmaps obtained from the pseudo-speckle analysis showing no substantial changes in retrograde flow. Arrows highlight region of lamellae overlap. (B) Kymograph of lamellar activity in colliding partners in a region perpendicular to the point of cell contact (red regions highlight lamellar retraction and blue extension). (C) The speed of lamellar retraction in *myosin II* mutants was quantified at the time of separation to reveal that the retraction rate was no different to freely moving cells. Error bars represent SD. *p < 0.05. Note that control retraction rates are from Figure 1J.

(D) Quantification of actin fiber formation in control, *zip*^{1} and *dia*^{5} mutant hemocytes during CIL. The graph represents the relative increase in actin intensity within the region encompassing the actin fiber (red box in schematic) with respect to the surrounding regions of the actin network (blue boxes in schematic).

(E) Quantification of the cessation of forward movement during CIL in which the mean distance between the initial point of contact and the nucleus was measured and compared to the distance at the time of cell separation. This analysis revealed that the *zip*^{1} and *dia*^{5} mutants failed to inhibit their forward motion in comparison to control cells. Error bars represent SD. *p < 0.05.

(F) Graph of mean time of lamellae contact revealed that *zip*^{1} and *dia*^{5} mutants maintained cell-cell contacts for a longer duration than control cells. Error bars represent S.D. **p < 0.01.

(G–I) *diaphanous* mutant (*dia*^{5}) collisions analyzed as in (A), (B), and (C).

See also Figures S4, S5, and S6 and Movie S6.

intervals (Figure S7A) suggesting their response was not as tightly coordinated as controls (Figure S1A). Furthermore, after microtubule alignment the *dia*^{5} mutant cells showed no obvious movement away from their colliding partners (Figures 7B and S7B). We also quantified the velocities 240 s after microtubule alignment, which corresponds to the time when both control and *dia*^{5} mutant cells have separated (Figure 6F). This revealed that while control cells migrated away from the collision, *dia*^{5} mutants showed no significant directional preference with respect to their colliding partners (Figure 7C). These data suggest that *dia*^{5} mutant hemocytes fail to actively repel each other.

We next determined how the alteration in cell repulsion in *dia*^{5} mutant hemocytes affected their ability to form an evenly spaced pattern during their developmental dispersal. Analysis of the average regions occupied by hemocytes during their dispersal revealed that control cells migrated within defined domains along the ventral surface of the embryo (Figure 7D). In contrast, *dia*^{5} mutant hemocytes showed an aberrant domain pattern (Figure 7D) and cells patrolled for greater distances within the embryo (Figures 7E and 7F), suggesting that they were less confined in their motility. *dia*^{5} mutant hemocytes also had a slight reduction in cell spacing as shown by their decreased nearest neighbor distances (median = 17.5 µm for controls and 15.8 µm for *dia*^{5}, $p < 0.05$) (Figures S7C and S7D; Movie S7). This suggests that precise repulsion dynamics are helping confine the migration of cells to defined regions within the embryo. Previous mathematical modeling of hemocyte dispersal suggested that tightly controlled CIL behavior was

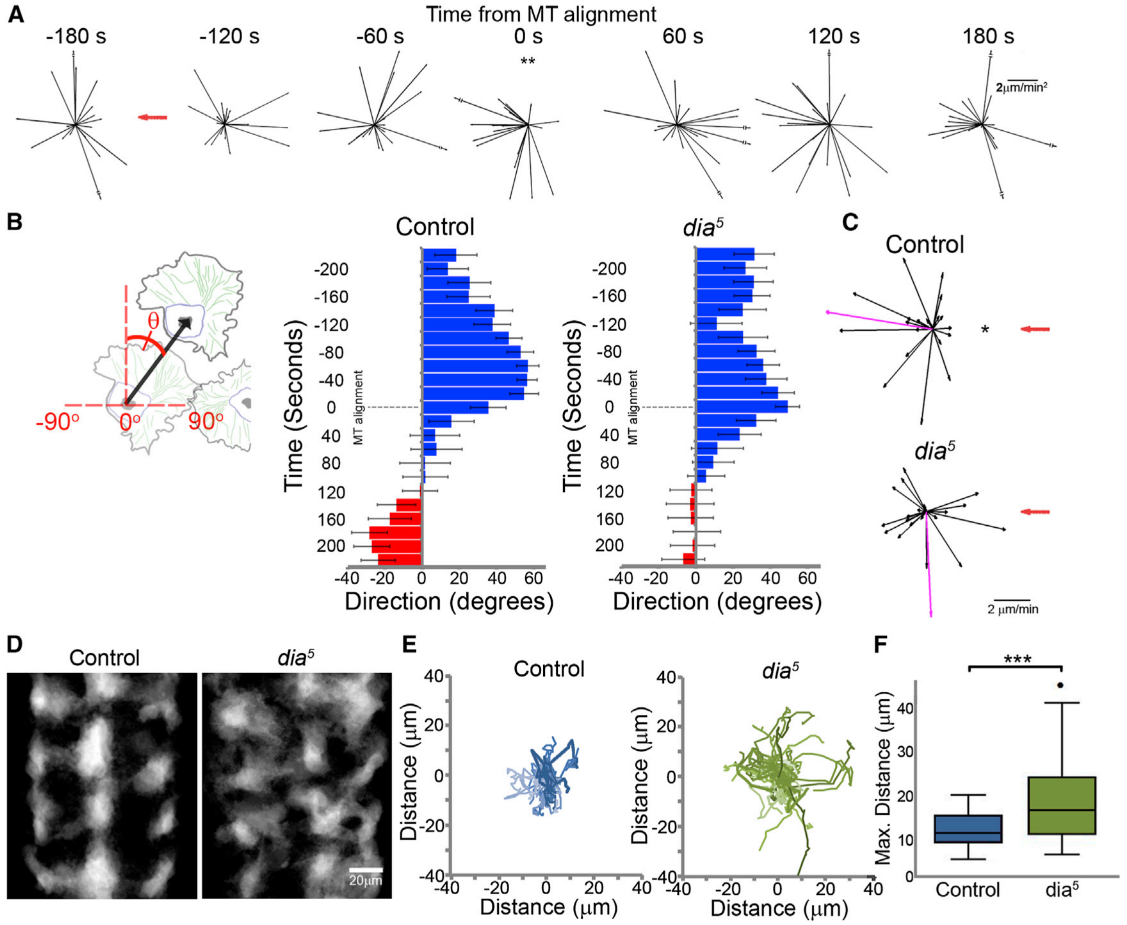

Figure 7. A Coordinated CIL Response Is Necessary Hemocyte Patterning

(A) Time course of hemocyte accelerations in *dia*^{5} mutants (black arrows) surrounding a collision event with reference to the colliding partner (red arrow). All time points show random accelerations except the time of microtubule alignment. $^{**}p < 0.01$.

(B) Quantification of average cell direction during the CIL time course as highlighted in the schematic. Blue highlights forward movement and red movement away from the colliding partner. Error bars represent SD.

(C) Cell velocities at 240 s after microtubule alignment with respect to the colliding partner (red arrow). Magenta arrows are the resultant velocities. Note that only controls show a significant movement away from the colliding partner. $^{*}p < 0.05$.

(D) The average regions occupied by hemocytes during their developmental dispersal revealed a disruption in the even spacing in *diaphanous* mutants.

(E) Tracks of hemocytes migrating over a 20-min period after they have spread throughout the embryo.

(F) Quantification of the maximum distance hemocytes migrate from the tracks measured in (E) revealed that *dia*^{5} mutants migrate over greater distances in the embryo. $^{***}p < 0.001$.

See also Figure S7 and Movie S7.

essential for their normal dispersal (Davis et al., 2012). As hemocytes in *dia*^{5} mutants showed no directional preference with respect to their colliding partners upon cell separation (Figure 7C), we wanted to determine how this reflected on the overall cell distribution in the simulation. Indeed, randomizing the sensitivity of simulated cells to the direction of their colliding partners (which in control simulations is a fixed parameter) led to a similar acquisition of aberrant domains (Figures S7E and S7F; Movie S7) and a slight reduction in cell spacing (median = 24.2 μm for wild-type parameters and 23.3 μm for randomized repulsion, $p < 0.001$). These data show that a precisely orchestrated CIL response in hemocytes is essential for it to behave as an efficient patterning cue.

DISCUSSION

Here, we show that hemocyte dispersal requires a precisely orchestrated CIL response; cells are not stochastically repelling each other. Hemocyte collision and subsequent repulsion involves a stereotyped sequence of kinematic stages that are modulated by synchronized changes in actin and microtubule dynamics. These integrated cytoskeletal changes are regulated by a transient inter-cellular adhesion, which physically couples the actin networks of colliding cells and builds up lamellar tension. It is this inter-cellular actin-clutch and the mechanical response to the collision that allows for a precise orchestration of cellular behaviors during CIL.

In recent years, there has been speculation that clutch-like mechanisms also exist at cell-cell junctions (Giannone et al., 2009). Indeed, engagement of cell-cell adhesion molecules in neuronal growth cones leads to similar slowing of actin retrograde flow (Schaefer et al., 2008). Furthermore, apical constriction during gastrulation, which is driven by acto-myosin contraction, is hypothesized to be induced by a "clutch-like" adhesion (Roh-Johnson et al., 2012). During CIL in hemocytes, this cell-cell adhesion is critical to orchestrate both intra-cellular responses and inter-cellular behaviors, which allows the response to be coordinated in colliding cells. As apical constriction during gastrulation is also coordinated in space and time across numerous cells within an epithelial sheet (Martin et al., 2010), it is possible that a similar clutch-like mechanism is allowing such inter-cellular integration of forces.

The engagement of a transient inter-cellular adhesion is characteristic of CIL in a number of cell-types (Gloushankova et al., 1998; Heaysman and Pegrum, 1973; Theveneau et al., 2010). This transient adhesion is very similar to the initial punctate adhesion between epithelial cells prior to their formation of a mature cell-cell junction, which also involves a radial actin bundle running perpendicular to the leading edge (Adams et al., 1998; Gloushankova et al., 1998). These transient cadherin-based adhesions, which have been called focal adherens junctions (Huveneers and de Rooij, 2013), depend on the development of tension (Huveneers et al., 2012). Indeed, cadherins in epithelial cells, astrocytes, and fibroblasts are observed to flow in a retrograde fashion with the actin network (Peglion et al., 2014), which has led to speculation that cadherin-based cell-cell adhesions could lead to an analogous clutch-like mechanism during maturation of adherens junctions (Giannone et al., 2009). It will be interesting to investigate why epithelial cells transform a focal adherens junction into a stable cell adhesion, whereas in fibroblasts and hemocytes the adhesion results in active repulsion.

Subsequent to adhesion engagement during CIL, we observe a sudden and synchronous reorganization of the colliding actin networks. The engagement of this actin-clutch develops tension between colliding cells, which we hypothesize causes the sudden forward acceleration that we observed immediately upon lamellar overlap. It is intriguing to note that chick heart fibroblasts similarly have a momentary acceleration toward each other during their CIL response (Abercrombie and Heaysman, 1953). This intracellular tension may also help form the transient stress fiber that couples the colliding cells, as stress fiber formation is a tension responsive process (Burridge and Wittchen, 2013). This creates a mechanism of haptic feedback whereby the cells "pull" on each other with the stress fiber acting as a mechanosensor during collisions, similar to its hypothesized role in sensing substrate stiffness (Trichet et al., 2012). The contraction of this actin fiber, which must be embedded within the lamellar actin network, would also explain the network-wide reorganization of actin flows in a process analogous to the "contractile treadmilling" observed toward regions of actomyosin contraction in fibroblasts (Rossier et al., 2010).

As the actin networks reorganize in colliding hemocytes, microtubules polymerize into the region of low retrograde flow (that also correlates with the region of stress fiber development). Microtubules polymerize toward the leading edge in a number of cell-types and undergo frequent catastrophes as they fight against the flowing actin network (Waterman-Storer and Salmon, 1997). In growth cones, upon adhesion engagement, the actin retrograde flow is slowed, allowing microtubules to polymerize toward the contact site (Schaefer et al., 2008) analogous to the initial stages of hemocyte CIL. We therefore speculate that the microtubule alignment observed between colliding cells during CIL is the result of microtubules following a path of least resistance within the actin network. However, it is also possible that microtubules are coupled to the stress fiber through an actin-microtubule crosslinker. Nevertheless, there must be tight coordination of both actin and microtubules during the CIL process.

While it is clear that microtubules are critical for CIL in a number of cell-types (Kadir et al., 2011; Stramer et al., 2010), their exact role during the response is currently unknown. The synchronous back acceleration in colliding partners is strongly correlated with the time when microtubule alignment occurs, suggesting that microtubule bundles are playing a role in stopping forward movement. The microtubules also appear to be critical for the generation of the precise internuclear spacing of the cells during CIL—that is critical for the emergence of the even dispersal pattern (Davis et al., 2012). Part of this spacing is governed by the regular size of hemocyte cell bodies; we hypothesize that the remainder of the nuclear spacing is controlled by the physical and dynamic properties of the microtubules themselves. As microtubules are rigid and capable of bearing compressive loads, the internuclear spacing may be controlled by a combination of tensional elements (i.e., the acto-myosin network) and elements that resist compression (i.e., microtubules). It may be the sum of these mechanical components—in a process analogous to cellular tensegrity (Ingber, 2003)—that allows the cells to precisely define their separation distance. Another possible role for microtubules may be in regulating the cell-cell adhesion. Microtubules target cell-matrix adhesions to promote their dissolution (Kaverina et al., 1999), and an analogous process may be occurring at the cell-cell adhesion during CIL.

Finally, the retraction phase of CIL involves a seemingly synchronous lamellar response in colliding partners. It is possible that the tension generated by engagement of the actin-clutch is suddenly released due to a breaking of the cell-cell adhesion. Alternatively, the tension may cause cytoskeletal components, such as the microtubule bundle or the actin stress fiber, to undergo a sudden catastrophe. Either way, it is this sudden release of lamellar tension that causes the synchronous retraction of colliding partners, which is essential to generate a choreographed CIL response. Lamellar retraction occurs prior to the generation of new protrusions away from the contact site, and we hypothesize that it is this sudden contraction that drives the subsequent repolarization phase of the response. Indeed, acto-myosin contractility initiates symmetry breaking and polarization in a number of cell-types (Cramer, 2010; Yam et al., 2007). It is also interesting to note that during initiation of polarized cell motility there is a propagation of actin network changes from the lamellar rear to the front (Yam et al., 2007), which we also observed during CIL initiation, although the mechanics behind this redistribution are currently unclear.

Here, we reveal that hemocyte CIL involves distinct stages, leading to both cells retracting from one another and subsequently repolarizing. It should be made clear that not all contact inhibitory cell-types undergo a similar sequence of events as CIL is not a homogenous response (Stramer et al., 2013). We favor the idea of broadly separating CIL into two types, first envisaged by Abercrombie (1970) and Vesely and Weiss (1973): type 1, which involves active retraction (e.g., hemocyte and fibroblast CIL), and type 2 in which forward movement is randomly deflected or stopped altogether (e.g., *dia*[5] mutant hemocytes, epithelial wound closure). While inhibition of cellular protrusions is sometimes thought to be the hallmark of CIL (Mayor and Carmona-Fontaine, 2010), Abercrombie (1970) believed that the predominant response in colliding cells is "a spasm of contraction" that "obliterates the process of ruffling." It is also interesting to note the initial description of CIL by Abercrombie and Heaysman (1953): "As a result of the adhesion…. they [cells] push or pull against each other to some extent; some of the energy which would normally go into movement is thereby dissipated or becomes potential energy of elastic tension between the cells. When an adhesion breaks, the release of potential energy stored as elastic tension produces the sudden acceleration." An inter-cellular actin-clutch is an ideal candidate to be responsible for such a response.

EXPERIMENTAL PROCEDURES

Microscopy

Embryos were mounted as previously described (Davis et al., 2012) and time-lapse images acquired every 5 s (for retrograde flow analysis) or 10 s (for kinematic and co-localization analyses) with a PerkinElmer Ultraview spinning disk microscope during developmental dispersal (stages 14–16). See Extended Experimental Procedures for a list of fly lines and a more detailed description of microscopy.

Kinematics Analysis and Modeling

For kinematic analysis, hemocytes were labeled with nuclear and microtubule markers and time-lapse movies were acquired at 10 s/frame. Nuclei were automatically tracked using Volocity software (PerkinElmer). Microtubule alignment was used as a marker for a CIL event, and cells that had not collided with another cell for 4 min before and after the microtubule alignment were included in the analysis. The velocity and acceleration of cells was calculated as previously described (Dunn and Paddock, 1982). See Extended Experimental Procedures for a more detailed description of kinematics.

Retrograde Flow Analysis

Time-lapse images of freely moving or colliding hemocytes containing actin labeled with either Lifeact-GFP or Moesin-cherry were acquired at 5 s/frame (when imaging actin alone) or at 10 s/frame (when imaging actin with other fluorescent probes). For collision analysis, cells were chosen such that the cells collided once over the duration of the time course. Cells were manually segmented prior to analysis. To quantify retrograde flow rates in lamellae, the cell body was manually segmented and data points within this region discarded. Pseudo-speckle analysis was performed as described previously (Betz et al., 2009). See Extended Experimental Procedures for a more detailed description of retrograde flow analysis.

Laser Abscission

Hemocytes were labeled with UAS-LifeAct-GFP and UAS-RedStinger and imaged on an inverted 780 Zeiss LSM multi-photon confocal with a time interval of 1 s. Cells were imaged for 5–10 s and then ablated with a two-photon laser tuned to 730 nm and focused in a 0.4 μm × 1.5 μm region, either at the edge or within the actin network for freely moving cells or at the region of lamella overlap along the actin fiber for colliding cells. Hemocytes were then imaged for 60 s with only 1.1 s elapsing between frames surrounding the ablation. For mock ablation of colliding cells, the same protocol was performed as mentioned except the laser was switched off. See Extended Experimental Procedures for a more detailed description of modeling lamellar forces.

Modeling Cytoskeletal Stresses

To compute the stresses inside the actin cytoskeleton in both freely moving and colliding hemocytes, the actin network was assumed to behave as a linear viscoelastic material. This work used the same model as Betz et al. (2011) to calculate the cytoskeletal forces developed by growth cones in vitro. See Extended Experimental Procedures for a more detailed description of modeling cytoskeletal stresses.

SUPPLEMENTAL INFORMATION

Supplemental Information includes Extended Experimental Procedures, seven figures, and seven movies and can be found with this article online at http://dx.doi.org/10.1016/j.cell.2015.02.015.

AUTHOR CONTRIBUTIONS

J.R.D., A.L., G.A.D., T.B., M.M., and B.M.S. contributed to the study concept and design, the analysis and interpretation of data, and the writing and revision of the manuscript. F.M., J.T., J.A.S., and Y.M. contributed to the acquisition and interpretation of data and review of the manuscript.

ACKNOWLEDGMENTS

We would like to thank M. Peifer and D. Kiehart for fly lines. We appreciate comments on the manuscript from B. Baum, G. Charras, N. Davis, M. Dionne, P. Martin, E. Paluch, and W. Razzell. We also would like to thank N. Tapon, and P. Jordan and D. Alibhai from LRI Light Microscopy for help with laser ablations. B.M.S. was supported by a Biotechnology and Biological Sciences Research Council (BBSRC) project grant BB/F020635/1. T.B. was supported by French Agence Nationale de la Recherche (ANR) grant ANR-11-JSV5-0002. J.R.D. was funded by the Medical Research Council (MRC). A.L. was funded by the BBSRC. We also thank the CMCBI at KCL for their support.

Received: March 14, 2014
Revised: November 24, 2014
Accepted: January 27, 2015
Published: March 19, 2015

REFERENCES

Abercrombie, M. (1970). Control mechanisms in cancer. Eur. J. Cancer *6*, 7–13.

Abercrombie, M., and Heaysman, J.E. (1953). Observations on the social behaviour of cells in tissue culture. I. Speed of movement of chick heart fibroblasts in relation to their mutual contacts. Exp. Cell Res. *5*, 111–131.

Adams, C.L., Chen, Y.T., Smith, S.J., and Nelson, W.J. (1998). Mechanisms of epithelial cell-cell adhesion and cell compaction revealed by high-resolution tracking of E-cadherin-green fluorescent protein. J. Cell Biol. *142*, 1105–1119.

Astin, J.W., Batson, J., Kadir, S., Charlet, J., Persad, R.A., Gillatt, D., Oxley, J.D., and Nobes, C.D. (2010). Competition amongst Eph receptors regulates contact inhibition of locomotion and invasiveness in prostate cancer cells. Nat. Cell Biol. *12*, 1194–1204.

Betz, T., Koch, D., Lim, D., and Käs, J.A. (2009). Stochastic actin polymerization and steady retrograde flow determine growth cone advancement. Biophys. J. *96*, 5130–5138.

Betz, T., Koch, D., Lu, Y.B., Franze, K., and Käs, J.A. (2011). Growth cones as soft and weak force generators. Proc. Natl. Acad. Sci. USA *108*, 13420–13425.

Burridge, K., and Wittchen, E.S. (2013). The tension mounts: stress fibers as force-generating mechanotransducers. J. Cell Biol. *200*, 9–19.

Carmona-Fontaine, C., Matthews, H.K., Kuriyama, S., Moreno, M., Dunn, G.A., Parsons, M., Stern, C.D., and Mayor, R. (2008). Contact inhibition of locomotion in vivo controls neural crest directional migration. Nature *456*, 957–961.

Cramer, L.P. (2010). Forming the cell rear first: breaking cell symmetry to trigger directed cell migration. Nat. Cell Biol. *12*, 628–632.

Davis, J.R., Huang, C.Y., Zanet, J., Harrison, S., Rosten, E., Cox, S., Soong, D.Y., Dunn, G.A., and Stramer, B.M. (2012). Emergence of embryonic pattern through contact inhibition of locomotion. Development *139*, 4555–4560.

Doyle, A.D., Kutys, M.L., Conti, M.A., Matsumoto, K., Adelstein, R.S., and Yamada, K.M. (2012). Micro-environmental control of cell migration—myosin IIA is required for efficient migration in fibrillar environments through control of cell adhesion dynamics. J. Cell Sci. *125*, 2244–2256.

Dunn, G.A., and Paddock, S.W. (1982). Analysing the motile behaviour of cells: a general approach with special reference to pairs of cells in collision. Philos. Trans. R. Soc. Lond. B Biol. Sci. *299*, 147–157.

Gardel, M.L., Shin, J.H., MacKintosh, F.C., Mahadevan, L., Matsudaira, P., and Weitz, D.A. (2004). Elastic behavior of cross-linked and bundled actin networks. Science *304*, 1301–1305.

Gardel, M.L., Sabass, B., Ji, L., Danuser, G., Schwarz, U.S., and Waterman, C.M. (2008). Traction stress in focal adhesions correlates biphasically with actin retrograde flow speed. J. Cell Biol. *183*, 999–1005.

Giannone, G., Dubin-Thaler, B.J., Rossier, O., Cai, Y., Chaga, O., Jiang, G., Beaver, W., Döbereiner, H.G., Freund, Y., Borisy, G., and Sheetz, M.P. (2007). Lamellipodial actin mechanically links myosin activity with adhesion-site formation. Cell *128*, 561–575.

Giannone, G., Mège, R.M., and Thoumine, O. (2009). Multi-level molecular clutches in motile cell processes. Trends Cell Biol. *19*, 475–486.

Gloushankova, N.A., Krendel, M.F., Alieva, N.O., Bonder, E.M., Feder, H.H., Vasiliev, J.M., and Gelfand, I.M. (1998). Dynamics of contacts between lamellae of fibroblasts: essential role of the actin cytoskeleton. Proc. Natl. Acad. Sci. USA *95*, 4362–4367.

Heaysman, J.E., and Pegrum, S.M. (1973). Early contacts between fibroblasts. An ultrastructural study. Exp. Cell Res. *78*, 71–78.

Hirata, H., Tatsumi, H., and Sokabe, M. (2008). Zyxin emerges as a key player in the mechanotransduction at cell adhesive structures. Commun. Integr. Biol. *1*, 192–195.

Homem, C.C., and Peifer, M. (2009). Exploring the roles of diaphanous and enabled activity in shaping the balance between filopodia and lamellipodia. Mol. Biol. Cell *20*, 5138–5155.

Hotulainen, P., and Lappalainen, P. (2006). Stress fibers are generated by two distinct actin assembly mechanisms in motile cells. J. Cell Biol. *173*, 383–394.

Huveneers, S., and de Rooij, J. (2013). Mechanosensitive systems at the cadherin-F-actin interface. J. Cell Sci. *126*, 403–413.

Huveneers, S., Oldenburg, J., Spanjaard, E., van der Krogt, G., Grigoriev, I., Akhmanova, A., Rehmann, H., and de Rooij, J. (2012). Vinculin associates with endothelial VE-cadherin junctions to control force-dependent remodeling. J. Cell Biol. *196*, 641–652.

Ingber, D.E. (2003). Tensegrity I. Cell structure and hierarchical systems biology. J. Cell Sci. *116*, 1157–1173.

Ji, L., Lim, J., and Danuser, G. (2008). Fluctuations of intracellular forces during cell protrusion. Nat. Cell Biol. *10*, 1393–1400.

Kadir, S., Astin, J.W., Tahtamouni, L., Martin, P., and Nobes, C.D. (2011). Microtubule remodelling is required for the front-rear polarity switch during contact inhibition of locomotion. J. Cell Sci. *124*, 2642–2653.

Kaverina, I., Krylyshkina, O., and Small, J.V. (1999). Microtubule targeting of substrate contacts promotes their relaxation and dissociation. J. Cell Biol. *146*, 1033–1044.

Martin, A.C., Gelbart, M., Fernandez-Gonzalez, R., Kaschube, M., and Wieschaus, E.F. (2010). Integration of contractile forces during tissue invagination. J. Cell Biol. *188*, 735–749.

Mayor, R., and Carmona-Fontaine, C. (2010). Keeping in touch with contact inhibition of locomotion. Trends Cell Biol. *20*, 319–328.

Nakano, K., Takaishi, K., Kodama, A., Mammoto, A., Shiozaki, H., Monden, M., and Takai, Y. (1999). Distinct actions and cooperative roles of ROCK and mDia in Rho small G protein-induced reorganization of the actin cytoskeleton in Madin-Darby canine kidney cells. Mol. Biol. Cell *10*, 2481–2491.

Peglion, F., Llense, F., and Etienne-Manneville, S. (2014). Adherens junction treadmilling during collective migration. Nat. Cell Biol. *16*, 639–651.

Roh-Johnson, M., Shemer, G., Higgins, C.D., McClellan, J.H., Werts, A.D., Tulu, U.S., Gao, L., Betzig, E., Kiehart, D.P., and Goldstein, B. (2012). Triggering a cell shape change by exploiting preexisting actomyosin contractions. Science *335*, 1232–1235.

Rossier, O.M., Gauthier, N., Biais, N., Vonnegut, W., Fardin, M.A., Avigan, P., Heller, E.R., Mathur, A., Ghassemi, S., Koeckert, M.S., et al. (2010). Force generated by actomyosin contraction builds bridges between adhesive contacts. EMBO J. *29*, 1055–1068.

Sandbo, N., Ngam, C., Torr, E., Kregel, S., Kach, J., and Dulin, N. (2013). Control of myofibroblast differentiation by microtubule dynamics through a regulated localization of mDia2. J. Biol. Chem. *288*, 15466–15473.

Schaefer, A.W., Schoonderwoert, V.T., Ji, L., Mederios, N., Danuser, G., and Forscher, P. (2008). Coordination of actin filament and microtubule dynamics during neurite outgrowth. Dev. Cell *15*, 146–162.

Stramer, B., Moreira, S., Millard, T., Evans, I., Huang, C.Y., Sabet, O., Milner, M., Dunn, G., Martin, P., and Wood, W. (2010). Clasp-mediated microtubule bundling regulates persistent motility and contact repulsion in Drosophila macrophages in vivo. J. Cell Biol. *189*, 681–689.

Stramer, B.M., Dunn, G.A., Davis, J.R., and Mayor, R. (2013). Rediscovering contact inhibition in the embryo. J. Microsc. *251*, 206–211.

Svitkina, T.M., Verkhovsky, A.B., McQuade, K.M., and Borisy, G.G. (1997). Analysis of the actin-myosin II system in fish epidermal keratocytes: mechanism of cell body translocation. J. Cell Biol. *139*, 397–415.

Theveneau, E., Marchant, L., Kuriyama, S., Gull, M., Moepps, B., Parsons, M., and Mayor, R. (2010). Collective chemotaxis requires contact-dependent cell polarity. Dev. Cell *19*, 39–53.

Trichet, L., Le Digabel, J., Hawkins, R.J., Vedula, S.R., Gupta, M., Ribrault, C., Hersen, P., Voituriez, R., and Ladoux, B. (2012). Evidence of a large-scale mechanosensing mechanism for cellular adaptation to substrate stiffness. Proc. Natl. Acad. Sci. USA *109*, 6933–6938.

Vesely, P., and Weiss, R.A. (1973). Cell locomotion and contact inhibition of normal and neoplastic rat cells. Int. J. Cancer *11*, 64–76.

Villar-Cerviño, V., Molano-Mazón, M., Catchpole, T., Valdeolmillos, M., Henkemeyer, M., Martínez, L.M., Borrell, V., and Marín, O. (2013). Contact repulsion controls the dispersion and final distribution of Cajal-Retzius cells. Neuron *77*, 457–471.

Waterman-Storer, C.M., and Salmon, E.D. (1997). Actomyosin-based retrograde flow of microtubules in the lamella of migrating epithelial cells influences microtubule dynamic instability and turnover and is associated with microtubule breakage and treadmilling. J. Cell Biol. *139*, 417–434.

Yam, P.T., Wilson, C.A., Ji, L., Hebert, B., Barnhart, E.L., Dye, N.A., Wiseman, P.W., Danuser, G., and Theriot, J.A. (2007). Actin-myosin network reorganization breaks symmetry at the cell rear to spontaneously initiate polarized cell motility. J. Cell Biol. *178*, 1207–1221.

Young, P.E., Richman, A.M., Ketchum, A.S., and Kiehart, D.P. (1993). Morphogenesis in Drosophila requires nonmuscle myosin heavy chain function. Genes Dev. *7*, 29–41.

Supplemental Information

EXTENDED EXPERIMENTAL PROCEDURES

Microscopy

Wild-type, UAS-Dia-CA, *zip*^{1} and *dia*^{5} mutant (Bloomington Stock Center) hemocytes were labeled using the promoters, *Srp*-Gal4 or *Sn*-Gal4. All crosses with the *dia*^{5} mutants were kept at 25°C as this allele is temperature sensitive (Homem and Peifer, 2009). The following fluorescent probes were used: nuclei (UAS-RedStinger); microtubules (UAS-Clip-GFP or UAS-Clip-Cherry) (Stramer et al., 2010); actin (UAS-Lifeact-GFP or UAS-Moesin-Cherry); Diaphanous (UAS-GFP-Dia) (Homem and Peifer, 2009); Myosin II heavy chain (UAS-GFP-Zip) (Franke et al., 2005); and Zyxin (UAS-Zyxin-Cherry) (Bloomington Stock Center).

For analyzing the formation of the actin fiber during collisions, the mean intensity of UAS-LifeAct-GFP was measured and normalized to the average intensity in a region 2.7 by 23.4 μm (approximately the length and width of the actin fiber across both cells) over the entire timecourse, n = 4 paired cells. For comparison of Myosin II and actin accumulation along the actin fiber, the mean intensity of UAS-GFP-Zip and UAS-LifeAct-GFP was measured in a region 2.7 by 11.7 μm (approximately the length and width of the actin fiber within the lamella of a single cell), n = 4 cells. To assess whether the actin fiber formed in *zip*^{1} and *dia*^{5} mutant hemocytes during collisions, an 8.1 by 11.7 μm region within the lamella perpendicular to the site of initial cell contact was manually cropped from each colliding partner. This cropped region was then separated into three rectangular areas each 2.7 by 11.7 μm, and the mean intensities measured at the time-point just before lamella separation. The ratio between the mean intensity of UAS-LifeAct-GFP in the central rectangular area (which encompassed the region of the actin fiber) and the average of the mean intensity in the two lateral rectangles was calculated. For statistical analysis a two-sample t-test was performed, n = 10 for control, 7 for *zip*^{1}, and 10 for *dia*^{5} hemocytes.

11 control collsions were analyzed to examine the time between lamella contact and microtubule alignment, microtubule alignment and cell separation, and total time in contact. The duration in contact of *zip*^{1} and *dia*^{5} hemocytes was also measured, n = 5 for *zip*^{1} and 11 for *dia*^{5} collisions. Mutant time in contact was compared to control collisions and statistically analyzed with a two-sample t test.

To track Myosin II particles in freely moving and colliding hemocytes, UAS-GFP-Zip puncta were manually tracked for 40 seconds. The displacement along the horizontal axis in freely moving and colliding hemocytes was statistically analyzed with a Mann-Whitney, n = 72 for freely moving cells and 77 for colliding cells.

For the extension-retraction analysis, segmented time-lapse images of hemocytes either freely moving or colliding were transformed in imageJ into binary images. Frames were either subtracted from the previous frame (to highlight retraction) or from the subsequent frame (to highlight extension). To compare lamellar activity in the cell front versus rear, the area of extension versus retraction was quantified in these regions of the cell and normalized to the average over the duration of the movie. To examine the rate of lamellar retraction, kymographs of LifeAct-GFP expressing hemocytes were generated perpendicular to the lamellar edge, and the distance over time was measured over a 5 second interval. All lamellar retraction events were quantified in freely moving cells over a period of 3 minutes and the average of these events used for subsequent analysis. For collisions, only the retraction event immediately after cell separation was quantified and used for subsequent analysis. For retraction speed, a two-sample t test with Welch's correction was performed to determine significance, for control hemocytes, n = 6 for collisions and 5 for freely moving cells; for *zip*^{1} hemocytes, n = 6 for collisions and 6 for freely moving cells; for *dia*^{5} hemocytes, n = 8 for collisions and 5 for freely moving cells.

To ascertain whether new protrusion formation preceded cell separation, the area of extension and retraction at the front and back of colliding hemocytes was measured and normalized to the average extension / retraction for the duration of the time-course, n = 8 cells.

Kinematics Analysis and Modeling

For statistical analysis of accelerations, a one-sample t test for the horizontal component was performed, n = 20 cells. To determine if control and dia^5 mutant hemocytes migrated away from their colliding partners 4 minutes after microtubule alignment, a bionomial test was performed on the unit vectors of the cell velocities (n=20 for both control and dia^5 mutant hemocytes).

For analysis on the internuclear distance and nuclear speed during collisions n = 20 for both control and *dia*^{5} mutant hemocytes.

To determine if hemocytes have any significant directional preference with respect to their colliding partners, we calculated the angle between the cell velocity and the horizontal axis. The velocity was computed with respect to the location of the colliding partner at 20 second intervals and then smoothed using a moving average window of 60 seconds. To eliminate any left or right bias, we normalized this angle between [-90, 90] degrees, where -90 degrees means that cells migrate in a direction opposite to their colliding partner, and 90 degrees signifies that hemocytes continued to move towards each other.

To analyze the cessation of forward movement upon lamellae contact we performed an assay in which the distance between the nuclei and initial point of contact was measured at the initial time of lamellae collision (D_t). This distance was once again measured but at the time of lamellae separation ($D_{t+\Delta t}$). The decrease in distance between the nuclei and point of contact during collisions was calculated using $D_{t+\Delta t} / D_t$. For statistical analysis on the cessation of forward movement assay we performed a two-sample t test with Welch's correction, n = 8 for control, 5 for *zip*^{1} and 7 for *dia*^{5} hemocytes.

In order to assess whether release of tension was sufficient to induce cell movement, cellular velocity was calculated 60 seconds after ablation in freely moving and colliding hemocytes. Cells were reorientated so that the ablation site was along the horizontal axis

and the average direction was calculated. We performed a binomial test on the unit vectors of the velocities to determine if there was a preferred forward or backward direction in cell movement with respect to the ablation site, n = 14 for freely moving; n = 12 for collisions which were ablated; and n = 16 for collisions which were mock ablated.

To measure the patterning dynamics of control and *dia*[5] hemocytes, cells were labeled with UAS-LifeAct-GFP and UAS-Red-Stinger and imaged for 90 minutes. To analyze the average position of hemocytes throughout the duration of this dispersal process, time-lapse images were thresholded into binary images and flattened as described by Davis et al. (2012). To quantify the dispersion of hemocytes, the nearest neighbor distance was measured in stage 16 embryos and a Mann-Whitney test performed to assess statistical significance, n = 324 cells from 9 control embryos and 309 cells from 9 *dia*[5] embryos. To characterize the migration dynamics of hemocytes in control and *dia*[5] embryos, cells were automatically tracked for 20 minutes. To assess the wandering nature of hemocytes the maximum distance away from the point of origin during the tracked time-course was measured, for statistical analysis a Mann-Whitney test was performed, n = 48 for both genotypes.

To compare the speed of control and *dia*[5] mutant hemocytes their mean displacement was calculated at minute intervals, n = 20 cells; additionally their directional ratio was measured over a 6 minute period at minute intervals, n = 8 cells for both genotypes.

The kinematic model of hemocyte dispersal was performed as described in Davis et al. (2012) and the collision sensitivity (ψ) [which determines how much a cell should take into account the direction of their colliding partners] was modified such that it could assume any value between [0, 1.25], thus randomizing their repulsion.

Retrograde Flow Analysis

Briefly, using a 2D cross-correlation algorithm adapted from classical particle velocimetry, this method compares a region of interest in an image (source image) with a subframe of a subsequent image (search image). To approximate the retrograde flow displacements, the algorithm tracked the motion of actin bundles and fluorescence intensity fluctuations within in the actin network.

For pseudo-speckle analysis, squares with an area of $1\mu m^2$ separated by $0.5\mu m$ from the source image were extracted, which corresponded to the size of the most prominent actin structures. The size of the search image was chosen such that it spanned the maximum expected displacement of the actin structures during the acquisition time, which in our case was 2.5 μm. To cover the whole search image, a cross-correlation coefficient was computed between source image and a sub-image of the search image shifted by one pixel. The displacement of the actin network was measured by finding the maximum coefficient within the resulting cross-correlation map.

To remove anomalous tracking data, only displacements that had a cross-correlation coefficient above a certain threshold, c_0, were kept. The value of this threshold was determined based on the quality of the images being analyzed, which was set to $c_0 = 0.5$ for the present work. As the retrograde flow has always been reported to be directed centripetally, all vectors that pointed within $\pm 45^\circ$ were discarded. Finally, a spatial convolution with a Gaussian kernel (variance of $1\mu m$), and temporal convolution with temporal kernel of 25 s were used to interpolate the measured displacements to cover all the pixels within the cell outline. The complete algorithm for this analysis, including the filtering and interpolation was implemented in MatLAB (MathWorks®).

To compute the average change in flow speed and average change in flow direction only data from the region of the cell outline that was common between two successive frames was used.

The change in flow direction, $\Delta\theta_{ij}$, was calculated using:

$$\Delta\theta_{ij}(t) = \tan^{-1}\frac{v_{ij}(t+1)}{u_{ij}(t+1)} - \tan^{-1}\frac{v_{ij}(t)}{u_{ij}(t)}, \quad [1]$$

where $\{u_{ij}(t), v_{ij}(t)\}$ represent the horizontal and vertical components of the flow vector at time point t at the location $\{i, j\}$ inside the cell outline.

To compare the flow rate in freely moving hemocytes the mean flow rate over a 3 – 4 minute period was measured in control, *zip*[1] mutant, *dia*[5] mutant and *zip*[1] rescued hemocytes (n = 6, 7, 6 and 4 respectively). For statistical analysis two-sample t tests were employed.

To investigate the flow rates and changes in flow direction during collisions, a region of 8.1 by 11.7 μm perpendicular to the site of initial cell contact was manually cropped from the lamella of each colliding partner, n = 8 cells for control and *dia*[5]. In each case, the area was chosen to avoid the region of lamellae overlap as the algorithm was unable to differentiate the overlapped colliding actin networks. To investigate the relationship between flow rates across colliding partners a cross-correlation of the instantaneous change in flow speed and direction within the cropped region was computed between colliding partners, n = 4 paired collisions for control and *dia*[5]. To investigate the spatial distribution of the observed retrograde flow changes during collisions, these regions were further divided into 3 equal boxes of 8.1 by 3.9 μm (representing the front, middle, and back of the lamella), and the average change in retrograde flow speed and direction were compared between the 3 smaller boxes, n = 8 cells.

For comparison of Zyxin intensity with actin network dynamics, the mean retrograde flow rate within a cropped area of the lamella that excludes the region of overlap (8.1 by 11.7 μm) perpendicular to the site of initial cell contact was calculated. To measure Zyxin intensity this region was extended to 8.1 by 16.3 μm in order to encompass the site of lamellae overlap where the adhesion was located. The maximum intensity of Zyxin was measured for the duration of the collision.

Modeling Lamella Stress

As recoil rate is representative of the tension within the actin network we performed kymography along the centre of the region of abscission in order to measure displacement for 12 seconds after ablation. In order to aid quantification of lamella displacement, kymographs were thresholded into binary images and the length from the region of ablation to the lamella measured. For comparison of initial recoil rates in freely moving and colliding hemocytes a two-sample t test with Welch's correction was performed to determine significance; n = 17 cells for collision and within the lamella of freely moving hemocytes regimes and n = 10 for the lamella edge of freely moving hemocytes.

The intra-lamelloipodial stresses were directly assessed using laser ablation experiments. Strain *u(t)* was calculated by dividing the initial deformation ΔI by the length of the lamella l. Both numbes were quantified from the kymographs as shown in Figure 4A of the main text. Retraction was analysed using a fast exponential relaxation overlaying the constant retrograde flow by fitting the function:$u(t) = u_0 \exp - (\omega t) + \nu t + o$. u_0 is the initial strain relaxation that we assume to be dominated by tension, ω is the relaxation frequency, ν the retrograde flow velocity and *o* an arbitrary offset. Subsequently, the force is calculated from the general mechanics relation: $F = E * u_0 * A$ by assuming the elastic modulus of E = 106 ± 21 Pa (see Betz et at., 2011) as explained below and the cross-sectional area A of 4μm^2, determined from the approximated height of the lamellipodium of 0.5μm and the lateral length of the recoiled part of 8μm. It is important to note that changing these parameters would only influence the magnitude and not the relative differences in force.

Modeling of Cytoskeletal Stresses

To estimate the stress (Figure 4E,F) developed inside the actin cytoskeleton in both freely moving and colliding hemocytes, the actin network was assumed to behave as a linear viscoelastic material.

It is important to note the model depends on the following network properties: the time dependent elastic modulus, relaxation time, viscosity, Poisson ratio, and Young's modulus, which are impossible to measure in hemocytes *in vivo*. However, as the lamellar morphology appeared similar in hemocytes and growth cones, and both have similar flow rates we therefore assumed that hemocytes have the same elastic properties as previously measured NG108 growth cones: relaxation time of τ_R = 2.4 ± 0.5 s, Young's modulus of E = 106 ± 21 Pa, viscosity of η = 905 ± 26 Pa s, Poisson ratio of ν = 0.47 ± 0.05 (Betz et al., 2011). As the model required integration of instantaneous stresses over the memory period of 40 seconds, and hemocyte edge movements were dynamic, stresses were only computed for the region of the cell that was common over this time period. Furthermore, during collisions, the actin cytoskeletons overlap resulting in the model assuming the lamellar networks are a continuous material. To verify that the observed redistribution of stresses is not an artifact of this assumption, cytoskeletal stresses were computed for a single cell segmented from a collision. This analysis revealed qualitatively similar results to the case when stresses were computed in both cells simultaneously suggesting that cytoskeletal stresses are indeed propagating from the back of the network towards the site of cell-cell contact.

SUPPLEMENTAL REFERENCES

Franke, J.D., Montague, R.A., and Kiehart, D.P. (2005). Nonmuscle myosin II generates forces that transmit tension and drive contraction in multiple tissues during dorsal closure. Curr. Biol. *15*, 2208–2221.

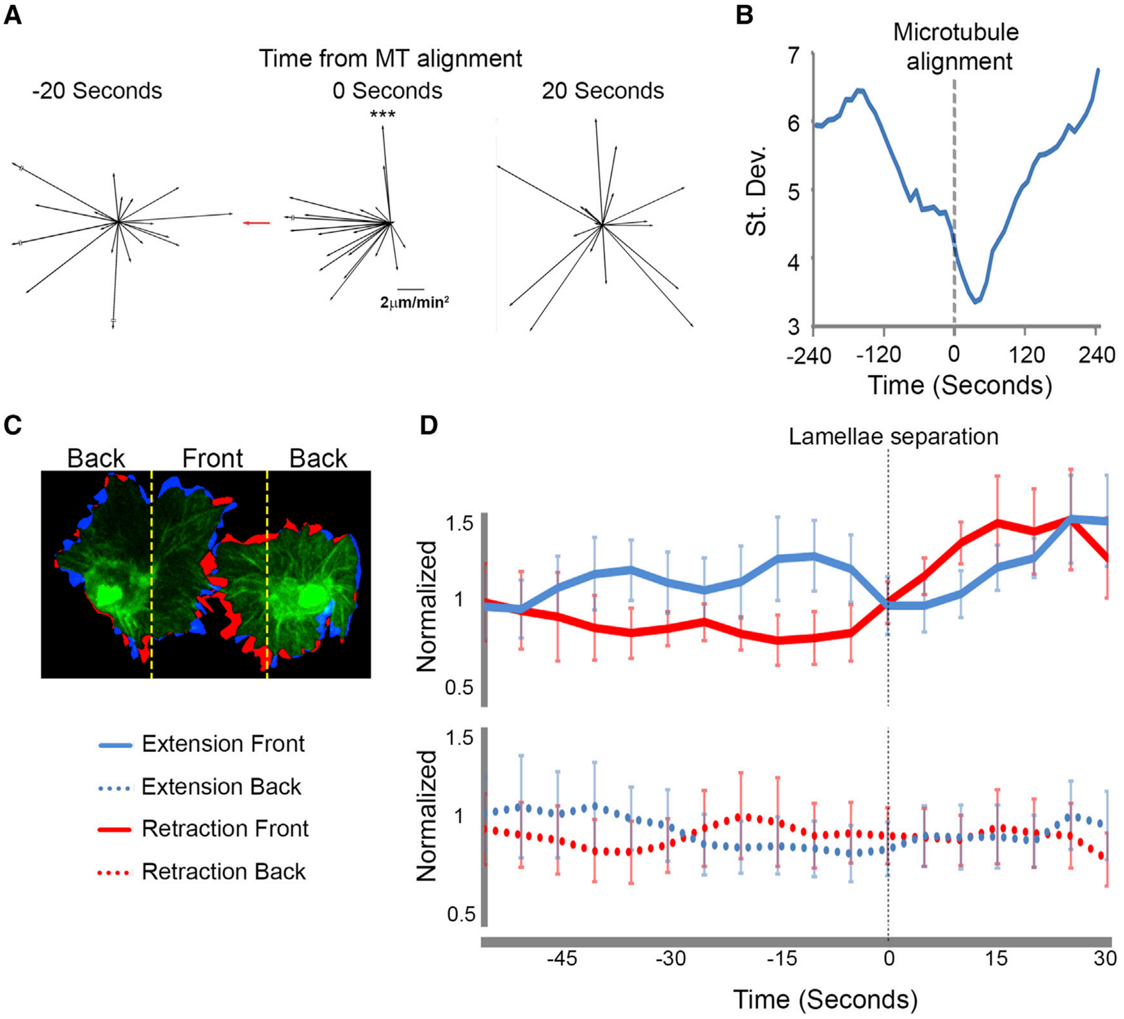

Figure S1. Hemocyte Contact Inhibition Is Precisely Controlled, Related to Figure 1

(A) Time course of hemocyte accelerations at 20 s intervals (black arrows) surrounding a collision event with reference to the colliding partner (red arrow). Note the significant back acceleration only upon microtubule alignment. ***$p < 0.001$.

(B) Plot of the SD from the analysis of internuclear distance over time during CIL (Figure 1D). Note that at −120 s the variance in the data decreased suggesting that the process was becoming regulated at this stage. Immediately upon microtubule alignment, the variance suddenly reduced again highlighting that microtubules were tightly controlling the spacing between colliding cells.

(C) Example of a hemocyte collision in which regions of lamellae extension (blue) and retraction (red) were highlighted. The lamellae of colliding cells were divided up into regions facing the colliding partner (front) or regions facing away from the colliding partner (back) and subsequently quantified.

(D) Average area of lamellar extension and retraction in the regions highlighted in (C). Note that during CIL, lamellar retraction at the front precedes new lamellae formation at the back of the cell. Error bars represent SEM.

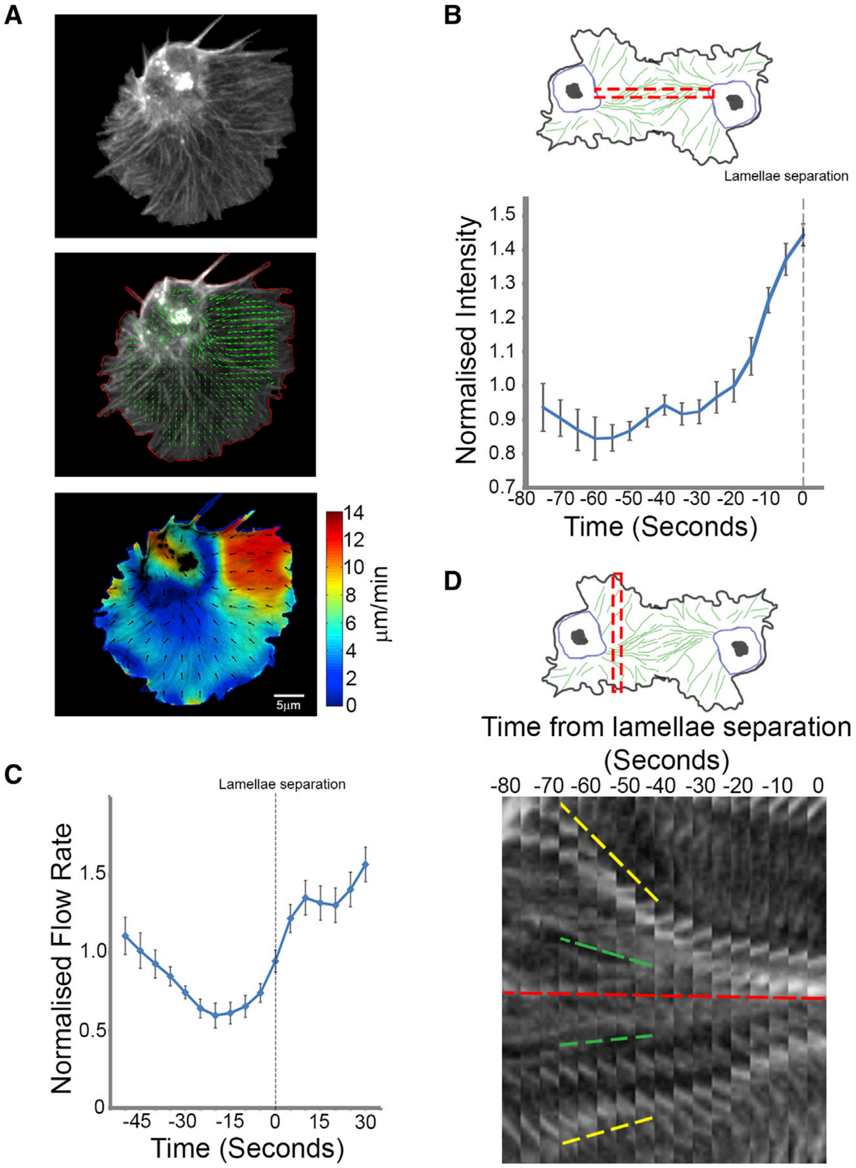

Figure S2. Actin Dynamics in Freely Moving and Colliding Hemocytes, Related to Figure 2

(A) A freely moving hemocyte containing labeled F-actin (top panel) analyzed by pseudo-speckle microscopy. Middle panel highlights the actin retrograde flow direction while the bottom panel shows both the magnitude and direction of the flow.

(B) Analysis of F-actin intensity within a corridor that corresponds to the region of actin fiber formation during contact inhibition as highlighted in the schematic.

(C) Analysis of the retrograde flow rate during CIL highlighting the reduction and subsequent increase in actin flow upon lamellae separation.

(D) Kymograph of a region perpendicular to the actin fiber (red dashed line in schematic) highlighting the recruitment of preexisting filamentous actin within the lamella. Note the increased rate of recruitment of actin fibers at the periphery (yellow dashed lines) compared with those closer to the center of the lamella (green dashed lines).

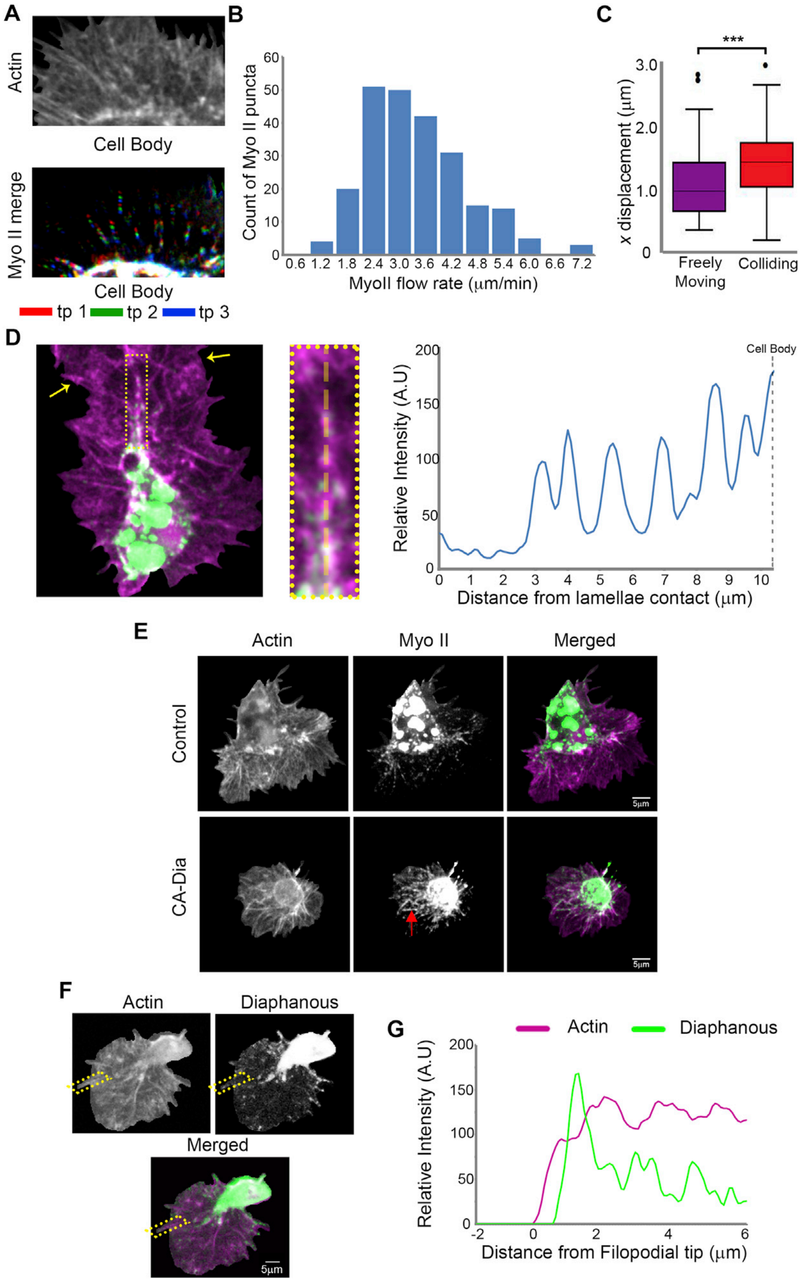

Figure S3. Myosin II and Diaphanous Localization in Hemocytes, Related to Figure 5

(A) Top panel shows a still image of a lamella of a freely moving hemocyte containing labeled F-actin. Bottom panel highlights tracked Myosin II particles within the lamella. The Myosin tracks were color coded in time to highlight their retrograde movement such that the first time point is labeled red, second time point green, and the third blue.

(B) Distribution of Myosin II particle speeds in freely moving hemocytes revealed a mean speed of 3.4 ± 0.6 μm/min.

(C) Quantification of the lateral displacement of Myosin II particles from the tracks in Figures 5B and 5D revealed an increase in horizontal displacement of Myosin II toward the actin fiber during cell collision. ***$p < 0.001$.

(D) Still image of a colliding hemocyte containing labeled F-actin (magenta) and Myosin II (green). Yellow arrows highlight the region of lamellae overlap. The inset shows the region of the actin fiber used for the line scan analysis. Note the repeating peaks of Myosin II intensity along the fiber.

(E) Still images of control and constitutively active Diaphanous expressing hemocytes with labeled F-actin (magenta) and Myosin II (green). Note the decrease in area of hemocytes containing constitutively active Diaphanous and the enhanced Myosin II localization within the lamella (arrow).

(F) Freely moving hemocyte containing labeled wild-type Diaphanous (green) and F-actin (magenta).

(G) Line scan of the region highlighted in (F) revealed a peak of Diaphanous at the ends of filopodia.

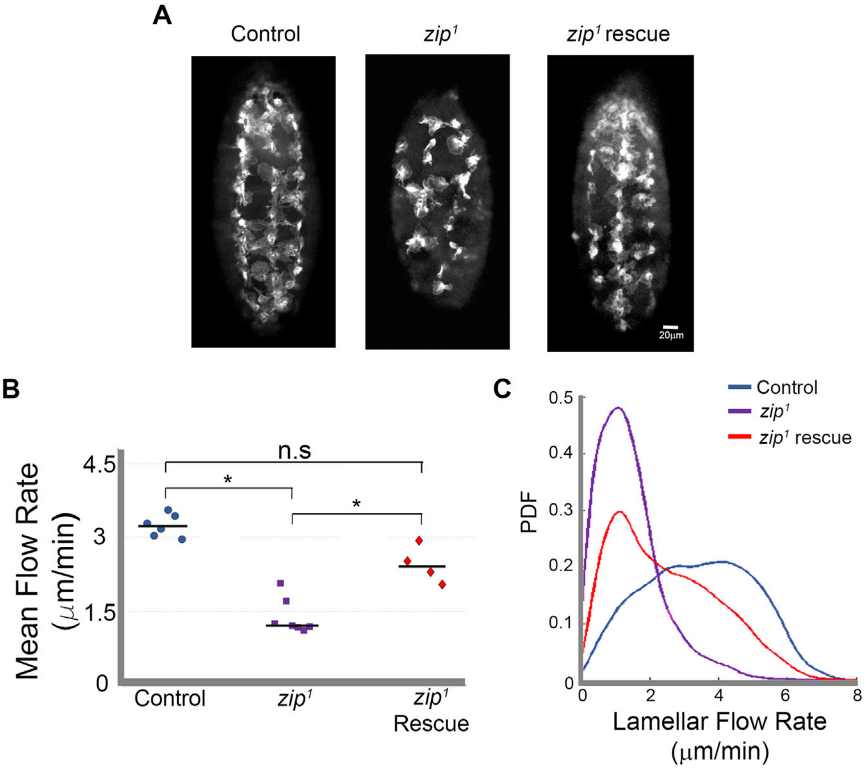

Figure S4. Myosin II Drives Retrograde Flow in Hemocytes, Related to Figure 6

(A) Still images of hemocyte dispersal along the ventral surface of Stage 15 embryos. Hemocyte dispersal was disrupted in *myosin II* mutant (*zip*^{1}) embryos and expression of GFP-tagged Myosin II specifically in *myosin II* mutants rescued dispersal.

(B) The mean retrograde flow across the lamella of freely moving cells was calculated for control, *myosin II* mutant (*zip*^{1}), and *myosin II* mutant rescue hemocytes. *$p < 0.05$. (C) Probability density function (PDF) of retrograde flow rates in freely moving control, *myosin II* mutant (*zip*^{1}), and *myosin II* mutant rescue hemocytes revealed that expression of GFP-tagged Myosin II increased the distribution of higher flow values in the *zip*^{1} mutant cells.

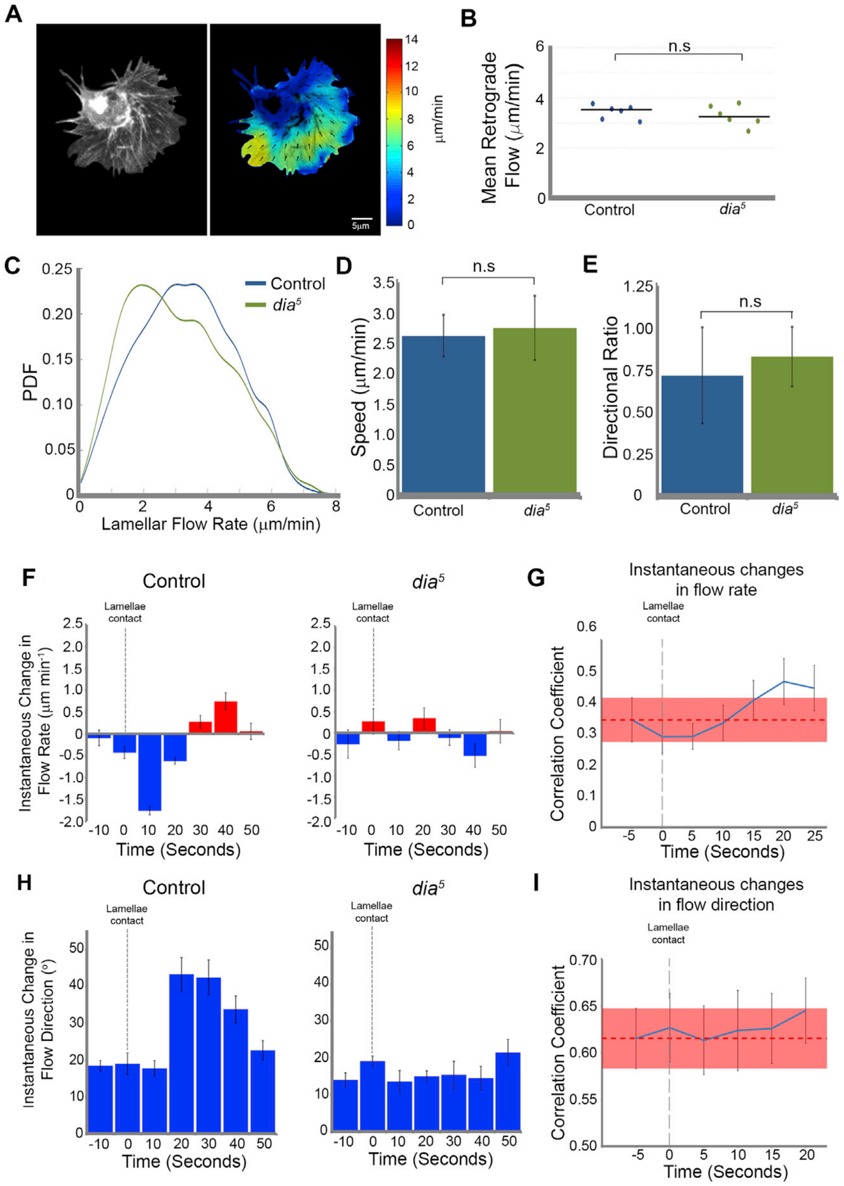

Figure S5. Migration of *diaphanous* Mutant Hemocytes, Related to Figure 6

(A) Left panel is a still image of a freely moving hemocyte containing labeled F-actin. Right panel is a heatmap of the actin retrograde flow field.

(B) The mean retrograde flow across the lamella of freely moving cells was calculated for control, and *diaphanous* mutant (*dia*^{5}) hemocytes.

(C) Probability density function (PDF) of retrograde flow rates in freely moving control and *diaphanous* mutant hemocytes revealed a similar distribution.

(D) Quantification of cell speed in freely moving control and *diaphanous* mutant hemocytes. Error bars represent SEM.

(E) Quantification of the directional ratio (persistence) of freely moving control and *diaphanous* mutant hemocytes. Error bars represent SEM.

(F) Quantification of instantaneous changes in flow rate in control and *diaphanous* mutant hemocytes during contact inhibition. Note that the control analysis was taken from Figure 2C. Error bars represent SD.

(G) Cross correlation of the instantaneous changes in flow rate in lamellae of colliding *diaphanous* mutant cells. Error bars represent SEM. Red dotted lines represent the mean correlation between colliding cells immediately prior to cell-cell contact with the thickness representing the SEM. Note that there is no increase in the correlation coefficient upon lamellae contact as observed in control cells (Figure 3G).

(H) Quantification of instantaneous changes in flow direction in control and *diaphanous* mutant hemocytes during contact inhibition. Note that the control analysis was taken from Figure 2E. Error bars represent SD.

(I) Cross correlation of the instantaneous changes in flow direction in lamellae of colliding *diaphanous* mutant cells. Error bars represent SEM. Red dotted lines represent the mean correlation between colliding cells immediately prior to cell-cell contact with the thickness representing the SEM. Note that there is no increase in the correlation coefficient upon lamellae contact as observed in control cells (Figure 3I).

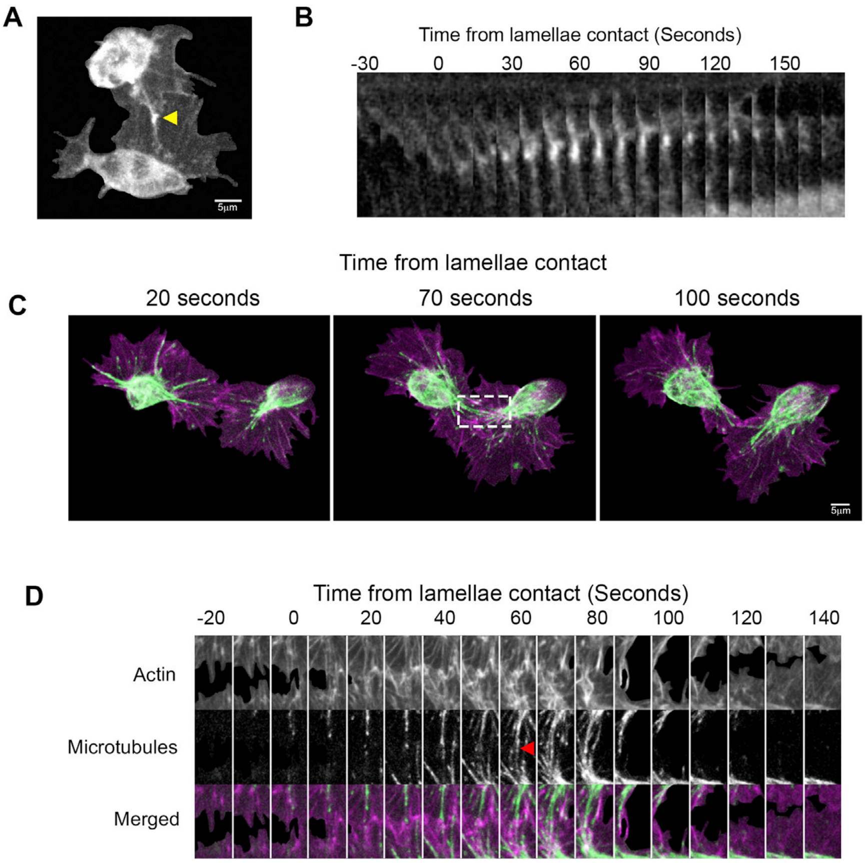

Figure S6. Analysis of Zyxin and Microtubule Dynamics in *diaphanous* Mutant Collisions, Related to Figure 6

(A) Localization of Zyxin in *diaphanous* mutant hemocytes during a collision. Note the puncta of Zyxin (arrowhead) at the region of lamellae overlap.

(B) Kymograph of Zyxin puncta during a *diaphanous* mutant collision. Note the presence of Zyxin at the region of lamellae overlap throughout the time course of the response.

(C) A collision between *diaphanous* mutant hemocytes containing labeled F-actin (magenta) and microtubules (green).

(D) Kymograph of the region highlighted in (C) showing the alignment between the microtubule networks (arrowhead) in *diaphanous* mutant cells.

Cell

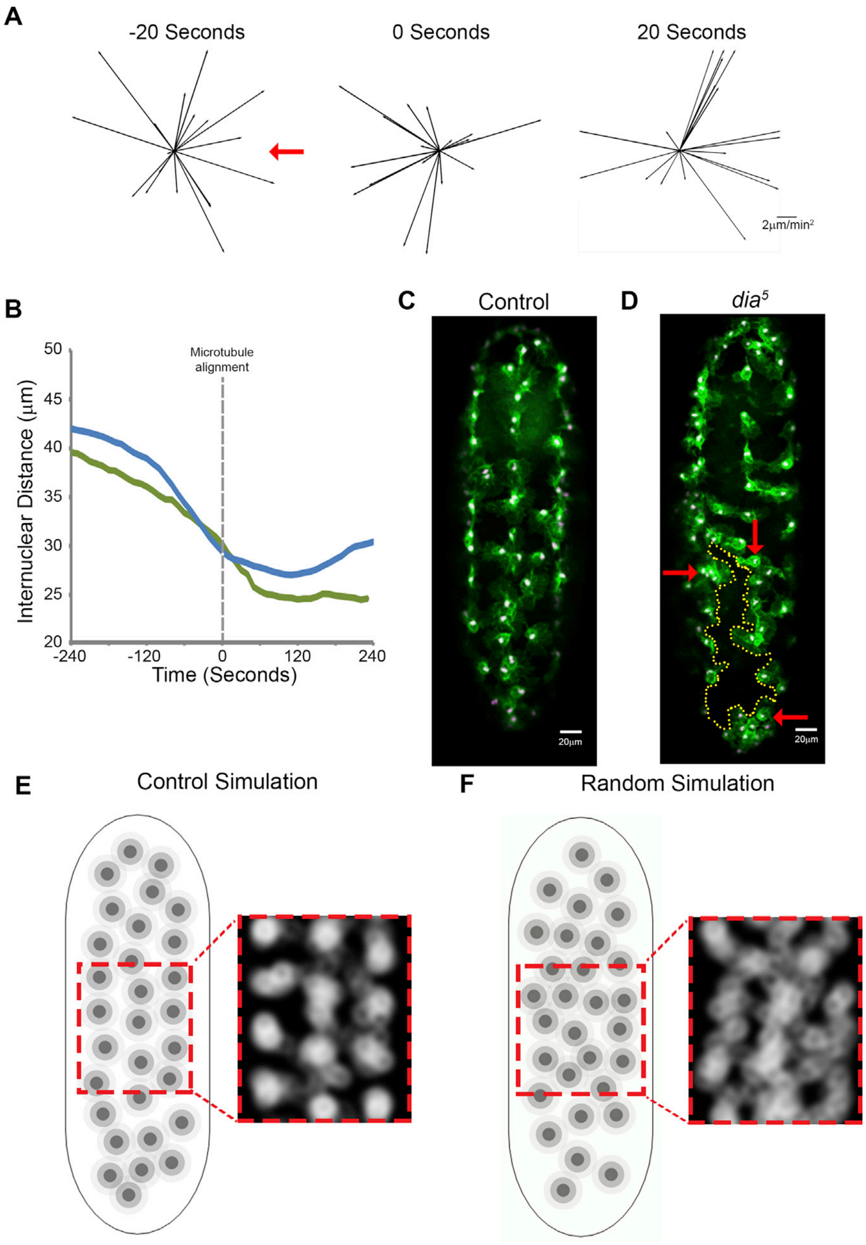

Figure S7. Aberrant CIL in *diaphanous* Mutant Cells Disrupts Hemocyte Spacing, Related to Figure 7

(A) Time course of *dia*^{5} mutant hemocyte accelerations at 20 s intervals (black arrows) surrounding a collision event with reference to the colliding partner (red arrow). Note that unlike controls (Figure S1A) there is no significant back acceleration upon microtubule alignment.

(B) Analysis of internuclear distance over time during the CIL response in control (blue line) and *diaphanous* mutant cells (green line). Note the reduced capacity of *diaphanous* mutant cells to slow down upon microtubule alignment and subsequently separate. Control analysis was taken from Figure 1D.

(C) Still image of wild-type hemocytes dispersed within the ventral surface of a Stage 15 *Drosophila* embryo containing labeled F-actin (green) and nuclei (magenta).

(D) Still image of *diaphanous* mutant hemocytes dispersed within the ventral surface of a Stage 15 *Drosophila* embryo containing labeled F-actin (green) and nuclei (magenta). Note the clumping of hemocytes (red arrows) with large regions devoid of cells (dotted line).

(E) Still image of a control simulation (as generated in Davis et al. [2012]) showing the even spacing of cells (left panel) which results in an evenly spaced domain map (right panel).

(F) Still image of a simulation in which the cells fail to consistently take into account in the direction of the colliding partner during CIL. Note the aberrant spacing of cells (left panel), which results in a failure to acquire a defined domain map (right panel).
